# Transcriptome-IPMS analysis reveals a tissue-dependent *miR156/SPL13* regulatory mechanism in alfalfa drought tolerance

**DOI:** 10.1186/s12864-020-07118-4

**Published:** 2020-10-19

**Authors:** Biruk A. Feyissa, Justin Renaud, Vida Nasrollahi, Susanne E. Kohalmi, Abdelali Hannoufa

**Affiliations:** 1 Department of Biology, University of Western Ontario, 1151 Richmond Street, London, Ontario N6A3K7 Canada; 2 Agriculture and Agri-Food Canada, 1391 Sandford Street, London, Ontario N5V 4T3 Canada

**Keywords:** Alfalfa, Drought, IPMS, *Medicago sativa*, miR156, SPL13, Transcriptome

## Abstract

**Background:**

We previously reported on the interplay between miR156/SPL13 and WD40–1/DFR to improve response to drought stress in alfalfa (*Medicago sativa* L.). Here we aimed to investigate whether the role of miR156/SPL13 module in drought response is tissue-specific, and to identify SPL13-interacting proteins. We analyzed the global transcript profiles of leaf, stem, and root tissues of one-month old RNAi-silenced *SPL13* (*SPL13*RNAi) alfalfa plants exposed to drought stress and conducted protein-protein interaction analysis to identify SPL13 interacting partners.

**Result:**

Transcript analysis combined with weighted gene co-expression network analysis showed tissue and genotype-specific gene expression patterns. Moreover, pathway analysis of stem-derived differentially expressed genes (DEG) revealed upregulation of genes associated with stress mitigating primary and specialized metabolites, whereas genes associated with photosynthesis light reactions were silenced in *SPL13*RNAi plants. Leaf-derived DEG were attributed to enhanced light reactions, largely photosystem I, II, and electron transport chains, while roots of *SPL13*RNAi plants upregulated transcripts associated with metal ion transport, carbohydrate, and primary metabolism. Using immunoprecipitation combined with mass spectrometry (IPMS) we showed that SPL13 interacts with proteins involved in photosynthesis, specialized metabolite biosynthesis, and stress tolerance.

**Conclusions:**

We conclude that the miR156/SPL13 module mitigates drought stress in alfalfa by regulating molecular and physiological processes in a tissue-dependent manner.

## Background

The frequent and extreme weather events in present-day are correlated with climate change, which aggravates crop losses [38, 42]. To cope with these weather events, plants respond by developing different resilience strategies at the phenotypic, physiological, and molecular levels [23, 51]. Among the molecular strategies, the role of microRNAs in regulating various plant processes to enhance stress tolerance has been reported in a variety of crops [21]. Of the hundreds of plant microRNAs, microRNA156 (miR156) is highly conserved across species and regulates plant development as well as tolerance to biotic and abiotic stresses [2, 4, 5, 15, 17, 21, 23, 35].

Recent findings showed moderate levels of *miR156* overexpression enhances drought tolerance in alfalfa by silencing *SPL13* [2] and enhancing the downstream *dihydroflavonol-4-reductase* (*DFR*) which mediates flavonoid biosynthesis [15]. Low to moderate levels of *miR156* and *WD40–1* overexpression [15], and silencing of *SPL13* [2] and *WD40–2* [3] resulted in drought tolerance with enhanced anthocyanin levels, and improved photochemistry and physiological adjustments. A recent plant microRNA profiling study revealed that the previously known 21 highly conserved microRNA families [49] were present in three alfalfa genotypes, in addition to 79 present in both alfalfa and *Medicago truncatula* [39]. Furthermore, two microRNAs (miR160 and miR408) were expressed in a tissue-specific manner, whereas six (miR156, miR159, miR166, miR319, miR396, and miR398) were abundantly expressed in all tissues [39].

Despite the conserved presence of miR156 in different tissues, previous findings showed that increased *miR156* expression affected alfalfa plant parts in different ways, including an increased number of lateral branches, improved root development, delayed flowering time and reduced plant height [4, 5, 17]. Hence, we hypothesize that the miR156/SPL13 module is regulated in a tissue-specific manner to orchestrate the necessary adjustments needed to cope with drought stress in alfalfa. We sought to investigate this hypothesis by using high throughput techniques of mRNA-based global transcriptomic analysis in leaf, stem, and root tissues of *SPL13*RNAi compared to empty vector alfalfa plants. Moreover, immunoprecipitation-based mass spectrometry (IPMS) combined with filter-assisted proteomics (FASP) was performed using *35S::SPL13-GFP* alfalfa plants to identify SPL13-interacting proteins that may contribute to drought stress.

## Results

**Fig. 1:**
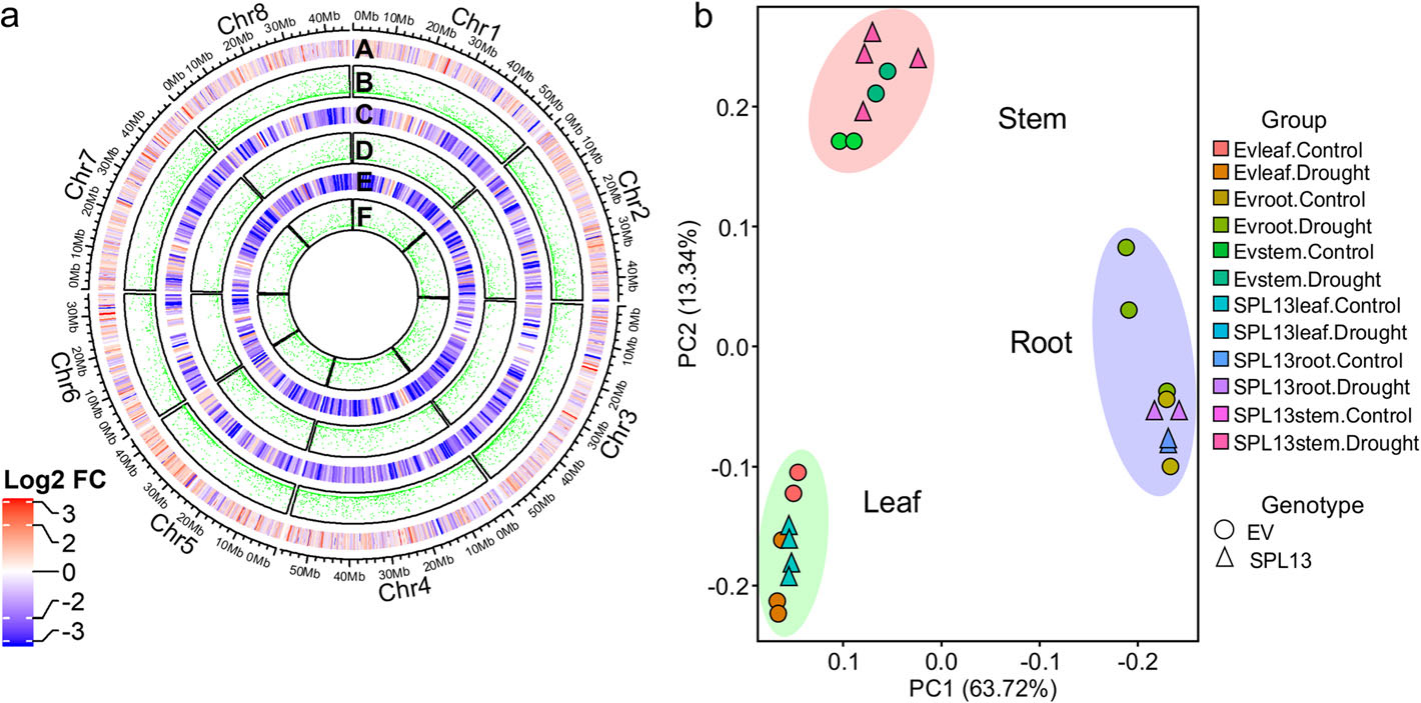
Principal Component Analysis (PCA) depicted tissue-dependent expression patterns. Circos plot (**a**) visualization of transcript fold changes and their significance levels in different tissues of alfalfa plants in response to drought stress. (A) Heat map of leaf-derived log2 transcript fold changes, (B) *p*_values of the DEG in leaf tissues, (C) Heat map of stem-derived log2 transcript fold changes, (D) *p*_values of the DEG in stem tissues, (E) Heat map of root-derived log2 transcript fold changes, (F) *p*_values of the DEG in root tissues. **b** PCA plots are constructed using total exon read counts from ‘.bam’ extension RNA sequenced samples irrespective of genotype and tissue. Transcript level comparisons in ‘a’ were between leaf, stem, and root tissues of drought-stressed SPL13RNAi and EV alfalfa plants. Red and blue colours from the heat map in ‘a’ represent an increased and decreased transcript log2 fold-changes, respectively. *P*_values are represented with green colours. Circlize (V 0.4.11), an R-software package was used for data visualization [19]. *n* = 3 biological replicates

To identify miR156/SPL13-regulated genes contributing to drought tolerance, high throughput transcriptomic analysis was conducted on alfalfa plants with reduced expression of *SPL13* (*SPL13*RNAi-65), compared to empty vector (EV) plants. On average, 7 million exon-region library sizes were generated from each replicate (Fig. S1a,b). Uniformly distributed DEG (corrected *p* value of *p* < 0.05 and an at least 2-fold changes) were observed across the eight chromosomes of the reference *Medicago truncatula* genome for each tissue type (Fig. 1a). Of the differentially expressed genes (DEG) derived from leaf, stem, and root tissues of drought-stressed *SPL13*RNAi and EV, more coverage was observed in leaf tissue followed by stem and root, respectively (Fig. 1a). The fold-changes from leaf tissues were greater towards increasing while stem and root tissues showed more decreased values in SPL13RNAi plants (Fig. 1a). Tissue-specific gene expression patterns were determined using total mRNA obtained from leaf, stem, and root tissues of these alfalfa genotypes (Fig. 1b). The major difference for the changes in transcript abundance is contributed by tissue type as explained by 63.7% of the variance in the Principal Component Analysis (PCA) (Fig. 1b).

### Genotype–specific transcript response of alfalfa to drought stress

**Fig. 2:**
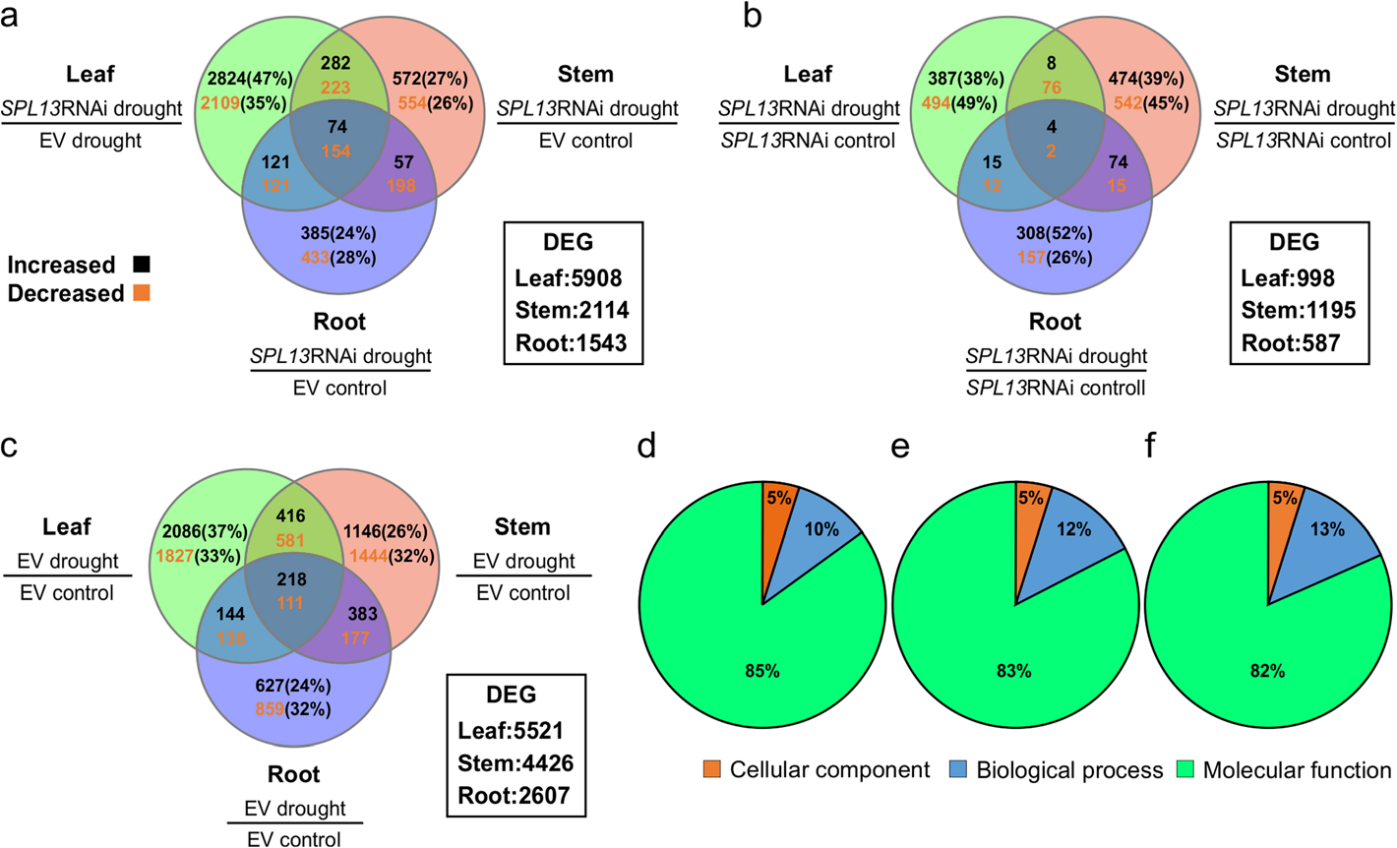
Tissue and genotype-specific expression patterns in genotypes of *SPL13*RNAi and EV alfalfa plants in response to drought. **a** Differentially expressed genes between drought stressed *SPL13*RNAi and EV plants; **b** *SPL13*RNAi-specific gene expression plasticity in response to drought stress; **c** EV-specific gene expression plasticity in response to drought stress; Gene Ontology (GO-term) –based percent representation of DEGs in cellular components, biological process, and molecular functions between *SPL13*RNAi and EV in **d** leaf, **e** Stem, and **f** Root tissues. The increased and decreased DEG percent provided in ‘a’, ‘b’, and ‘c’ vendiagramm were calculated from the total DEG of the specific tissue comparison. Upper and bottom values in ‘a’, ‘b’, and ‘c’ vendiagramm represent the number of significantly increased and decreased genes, respectively

Previous reports showed that the miR156/SPL13 module regulates physiological, metabolic and phenotypic adjustments in alfalfa under drought [2, 15]. To understand SPL13-driven drought stress tolerance strategy, the global transcriptomic profile of leaf, stem, and root tissues of RNAi-silenced SPL13 and EV plants were investigated under drought stress. A total of 5908, 2114, and 1543 DEG were found in leaf, stem, and root tissues, respectively (Fig. 2a). Of the DEG, 74 were commonly increased in *SPL13*RNAi plants regardless of tissue source, while 154 transcripts were commonly decreased (Fig. 2a). A list of the DEG is available in the supplementary file Table S1. Among the commonly increased 74 genes, the highest fold-change corresponds to vacuolar ion transporter-like protein (Medtr2g008110) followed by gibberellin-regulated family protein (Medtr6g007897), fasciclin-like arabinogalactan protein (FLAP) (Medtr5g098420), proline dehydrogenase (PDH) (Medtr7g020820), Pmr5/Cas1p GDSL/SGNH-like acyl-esterase family protein (Medtr4g079700), LRR receptor-like kinase (Medtr5g090100), and abscisic acid receptor (Medtr7g070050) (Table S1). Contrariwise, ABC transporter family protein (Medtr2g095440), plasma membrane H+ ATPase (Medtr3g108800), and PLAT-plant-stress protein (Medtr3g087490) were among the commonly reduced 154 genes in *SPL13*RANi plants under drought, irrespective of tissue source (Table S1).

To understand the drought-responsive transcript plasticity reflected by genotype, transcripts of drought-stressed and non-stressed tissues of *SPL13*RNAi and EV plants were analyzed. It was determined that *SPL13*RNAi had 998, 1195, and 587 DEG, whereas a considerably higher number (an average 4.5 fold) of DEG were observed in EV plants with 5521, 4426, and 2607 DEG in leaf, stem and root tissues, respectively (Fig. 2b,c; Table S2, S3).

### Leaf-specific transcript profile of alfalfa plants under drought

To understand the gene co-expression pattern upon drought stress across tissues and genotypes, exon read-counts from both genotypes and all tissues were subjected to weighted gene co-expression network analysis (WGCNA) (Fig. S2c) followed by principal component analysis (PCA) (Fig. 1**)**. Transcript profiles from leaf, stem, and root tissues of *SPL13*RNAi and EV responded to drought in a tissue-specific manner; presenting different clusters based on tissue type (Fig. 1). Of the 5908 DEG present between drought-stressed *SPL13*RNAi and EV leaf tissues, 47% of the genes were significantly increased and were leaf-specific in *SPL13*RNAi plants (Fig. 2a). On the other hand, considering tissue plasticity between well-watered and drought-stressed leaf tissues, 38% (of the 998) of DEG were increased in *SPL13*RNAi leaves (Fig. 2b) while 37% (of 5521) of DEG were increased in EV leaves (Fig. 2c). Moreover, 49% (494 of 998) of leaf-specific DEG were decreased in *SPL13*RNAi leaf tissues while 33% (1827 of 5521) were decreased uniquely in EV leaves (Fig. 2b,c). GO-terms of the DEG were analyzed and categorized into molecular function, biological process, and cellular components to understand the role of DEG between leaf tissues of drought-stressed *SPL13*RNAi and EV plants. GO-term analysis from leaf tissues showed 85% corresponding to molecular function followed by 10 and 5% to biological process and cellular components, respectively (Fig. 2d). Graphical representation of the components of GO-term analysis is provided in supplementary file Fig. S3. The top three highly represented leaf-derived GO-terms for the molecular function category correspond to transcription activity (phosphorelay response regulator activity, sequence-specific DNA binding transcription factor activity, and transcription cofactor activity) (Table S4.1). Likewise, the most highly represented three biological processes correspond to telomere maintenance, translation and alcohol metabolic process in addition to glutamine catabolic process and porphyrin-containing compound biosynthetic process (includes chlorophyll biosynthesis) (Table S4.1).

**Fig. 3:**
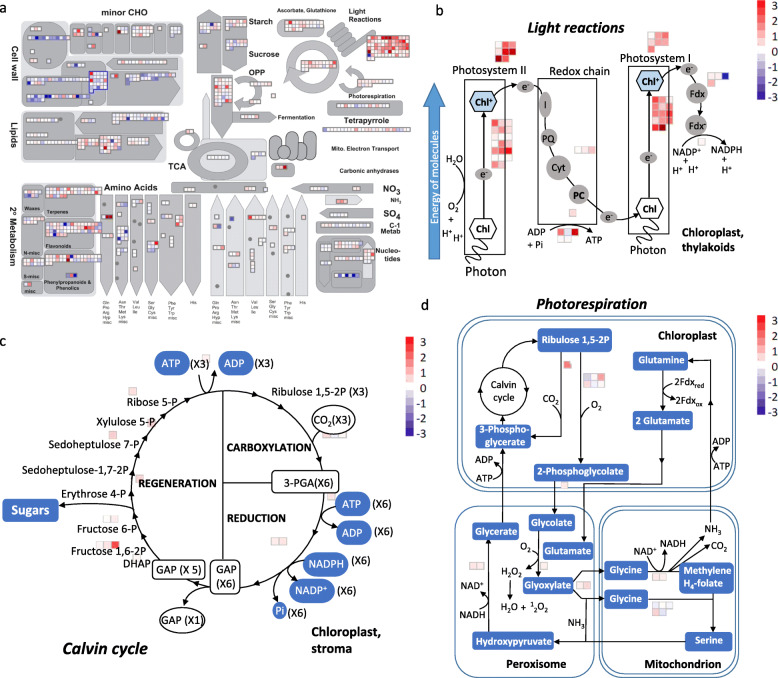
Leaf-specific DEG attributed to photosynthesis are enhanced in *SPL13*RNAi plants. **a** Summary of affected metabolites and pathways between drought-stressed EV and *SPL13*RNAi leaf tissues, **b** transcripts coding for light-dependent photosynthetic reaction in the chloroplast thylakoids, **c** carbon dioxide fixation in Calvin cycle in chloroplast stroma region, **d** photorespiration-associated transcripts involving chloroplast, mitochondria and peroxisome differentially regulated between drought-stressed *SPL13*RNAi and EV plants. Transcript fold changes are provided in log 2 with red and blue colours representing increased and decreased transcript levels relative to EV. Minor CHO corresponds to minor charbohydrate; TCA, Tricarboxylic acid cycle; OPP, oxidative pentose phosphate pathway. *N* = 3 biological replicates for each genotype and treatment condition

The differentially regulated genes were mapped to the *M. truncatula* genome using MapMan pathway analysis tool to understand their functional associations. Accordingly, leaf-specific DEG between drought-stressed *SPL13*RNAi and EV showed that various metabolic pathways were significantly affected, including carbohydrate metabolism, photosynthesis (mainly light reaction), and primary metabolism-related genes (Fig. 3a). Most importantly, photosynthesis-associated transcripts were highly increased in *SPL13*RNAi plants (Fig. 3a). Further investigation of transcripts associated with photosynthesis revealed that light-dependent reaction centers, namely of photosystem I and photosystem II, were expressed higher in *SPL13*RNAi (Fig. 3b). Unlike the light-dependent reaction centers, the Calvin cycle (Fig. 3c) and photorespiration-associated transcripts (Fig. 3d) were either slightly increased or not altered. Specifically, the carboxylation- and photorespiration-associated transcript of Rubisco (Ribulose-1,5-bisphosphate carboxylase/oxygenase) was slightly increased in *SPL13*RNAi plants under drought stress (Fig. 3c,d).

### Stem-specific transcript changes in alfalfa plants under drought

Expression levels of 27% of the 2114 DEG between drought-stressed stem tissues of *SPL13*RNAi and EV plants were increased exclusively in stems of *SPL13*RNAi plants (Fig. 2a). On the other hand, genotype- and stem-specific drought stress-responsive transcript plasticity revealed 39% of the 1195 DEGs in *SPL13*RNAi plants were increased while EV plants showed a 26% increase out of 4426 DEGs (Fig. 2b,c). Comparable to DEG in leaf tissues, the majority (83%) of the DEG in stem are associated with molecular functions followed by biological processes (12%) and cellular components (5%), despite DEG profile composition differences between leaves and stems (Fig. 2e; Fig. S4). The most represented three molecular functions which are differentially affected between *SPL13*RNAi and EV stem tissues are acyl-CoA dehydrogenase activity, ubiquinol-cytochrome-c reductase activity, and hydroxymethylglutaryl-CoA reductase (NADPH) activity (Table S4.2). On the other hand, the highly enriched categories of the affected biological processes include ATP catabolic process, response to stress, defense response, intercellular signal transduction and response to desiccation (Table S4.2).

**Fig. 4:**
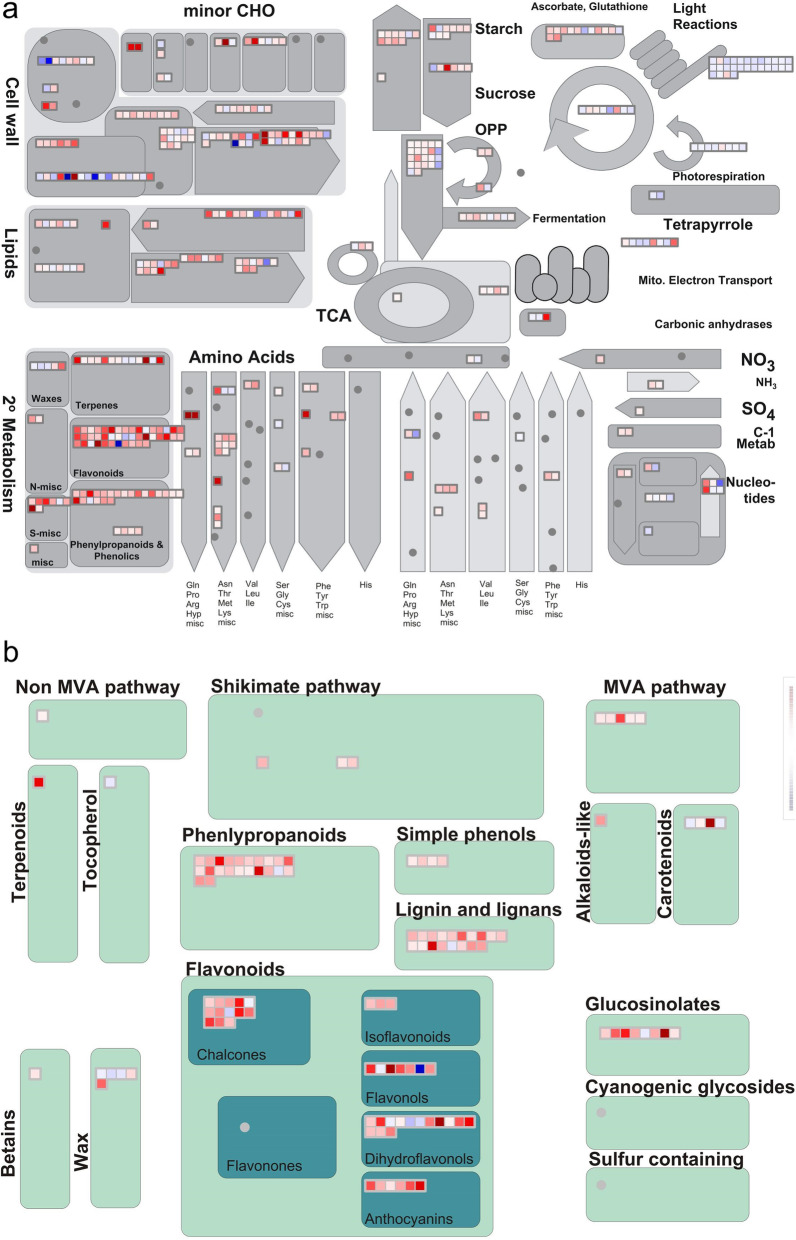
Stem-specific DEGs are associated with secondary metabolism. **a** Summary of affected metabolites and pathways between drought stressed EV and *SPL13*RNAi stem tissues, **b** distribution of the differentially expressed genes associated with secondary metabolism. Pathway analysis was performed using MapMan. Transcript fold changes are provided in log 2 with red and blue colours representing an increased and decreased transcript levels relative to EV. Minor CHO corresponds to minor charbohydrate; TCA, Tricarboxylic acid cycle; OPP, oxidative pentose phosphate pathway; MVA, mevalonate pathway. *N* = 3 biological replicates for each genotype and treatment condition

Furthermore, to understand the association of DEG of stem with metabolic pathways, the DEG were subjected to MapMan-based pathway analysis (Fig. 4a). DEG of stem tissues corresponded mainly with increased flavonoid biosynthesis, carbohydrate metabolism and response to desiccation in *SPL13*RNAi plants (Fig. 4a, Table S1). On the other hand, DEG associated with photosynthesis were decreased significantly in *SPL13*RNAi plants (Fig. 4a). Transcriptomic analysis of DEG obtained from stem tissues combined with MapMan-based pathway analysis revealed an activation of the phenylpropanoid pathway in *SPL13*RNAi plants under drought stress (Fig. 4b).

### Root-specific transcript profile of alfalfa plants under drought

**Fig. 5:**
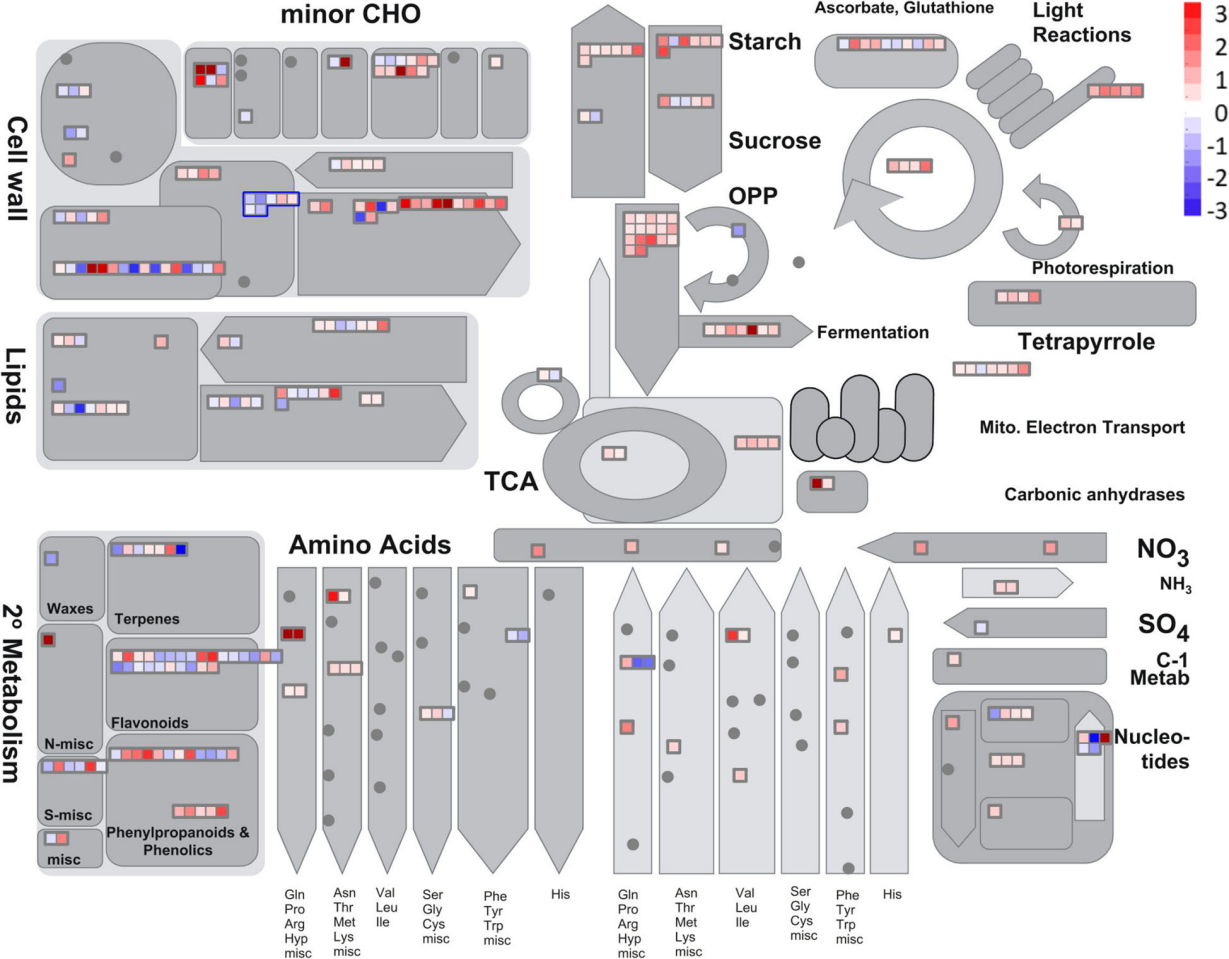
Distribution of root-specific differentially expressed genes between EV and *SPL13*RNAi plants. Summary of differentially affected metabolites and pathways between drought stressed EV and *SPL13*RNAi root tissues. Transcript fold changes are provided in log 2 with red and blue colours representing increased and decreased transcript levels relative to EV. Minor CHO corresponds to minor charbohydrate; TCA, Tricarboxylic acid cycle; OPP, oxidative pentose phosphate pathway. *N* = 3 biological replicates for each genotype and treatment conditions

A total of 1543 DEG were detected between roots of drought-stressed *SPL13*RNAi and EV plants, with 24 and 28% of them increased and decreased, respectively, exclusively in the roots (Fig. 2a). Further analysis of the plasticity between well watered and drought-stressed root samples showed 52% of 587 DEG in *SPL13*RNAi roots were upregulated while 24% of 2607 DEG were increased only in EV roots (Fig. 2b,c). Moreover, GO-term analysis of the root-specific DEG showed a similar proportion of components to that of stem and leaf tissues where the majority (82%) of transcripts belong to molecular function followed by biological process (13%) and cellular components (5%), but with a varied profile composition (Fig. 2f; Fig. S4). The top highly represented biological processes encompass ATP catabolic process, response to stress, defense response, intercellular signal transduction, phosphorelay signal transduction system, metabolic process, metal ion transport and transmembrane transport (Table S4.3). On the other hand, the major representation from molecular functions are attributed to phosphorelay response regulator activity, sequence-specific DNA binding transcription factor activity, catalytic activity, GTPase activity, secondary active sulfate transmembrane transporter activity (Table S4.3). Moreover, to further understand the DEG association, the DEG were subjected to MapMan-based pathway analysis. We found that metal ion transport, carbohydrate and primary metabolism were significantly and differentially affected between *SPL13*RNAi and EV plants in response to drought (Fig. 5). Moreover, cell wall and lipid biosynthesis were increased in roots of *SPL13*RNAi plants as compared to EV.

### Identification of SPL13 interacting proteins in alfalfa

**Fig. 6:**
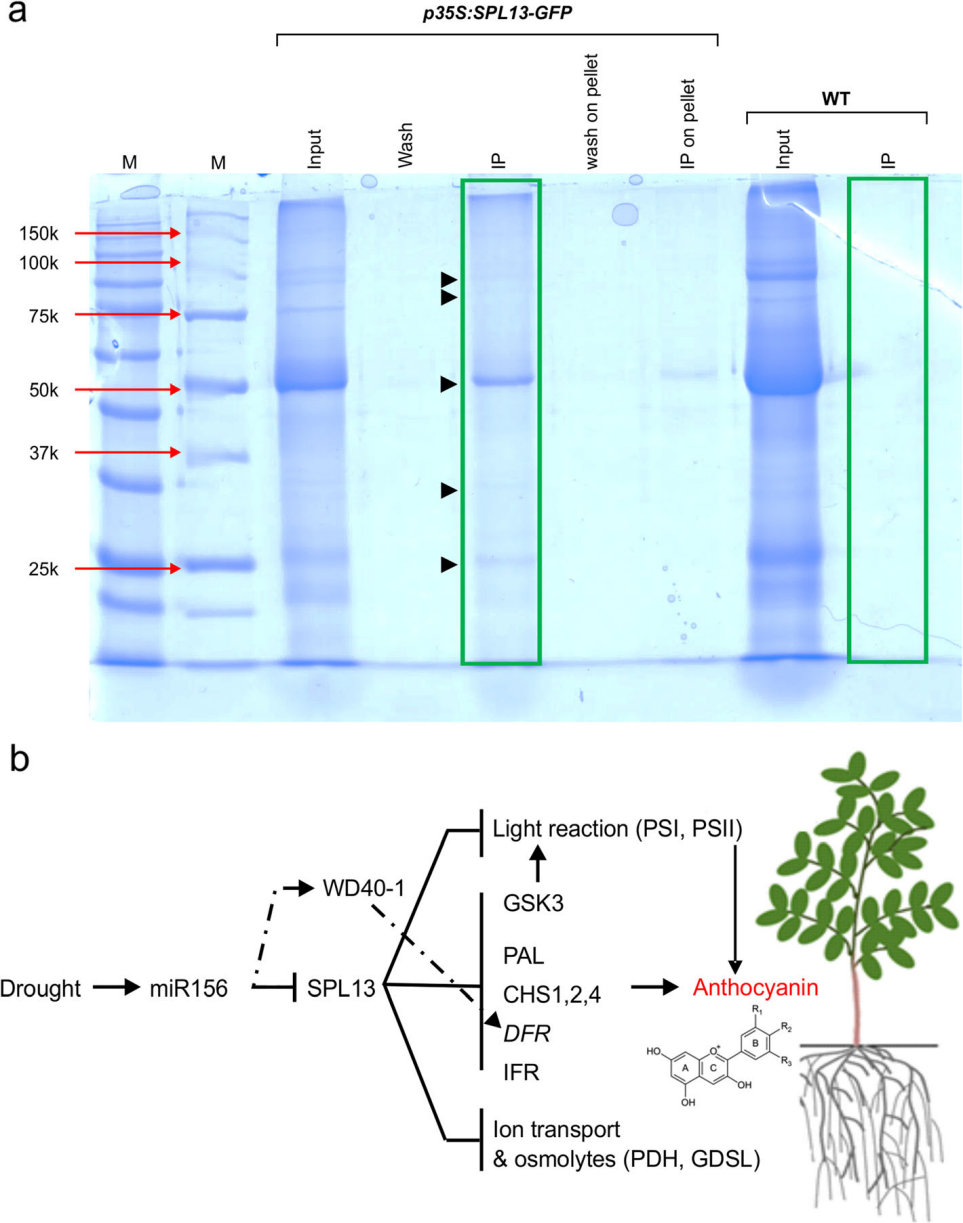
SPL13 interacts with proteins involved in photosynthesis process, stress alleviation, and specialized metabolite biosynthesis. **a** Coomasie stained SDS-PAGE gel, **b** a proposed tissue-specific drought tolerance model modulated by miR156/SPL13. The diagrammatic representation shows a tissue-specific miR156/SPL13 regulation module in response to drought tolerance. *miR156* is induced by drought stress, which in turn silences *SPL13* [2]. Reduced expression of *SPL13* driven by *miR156* and enhanced level of WD40–1 enhances *DFR* [15], together with less inactivation of GSK3 signalling with PAL, CHSs, and DFR result in accumulation of anthocyanins mainly in stem tissues. In moderate *miR156*OE plants, primary metabolites such as GABA, proline and sugars also accumulate for carbon-to-nitrogen balance and osmotic homeostasis. Induction of *miR156* during drought stress also enhances phenotypic plasticity, such as longer roots and higher biomass to access more water from the rhizosphere. With reduced *SPL13* expression and *miR156*OE, higher photosynthesis efficiency is also achieved during drought stress. The most prominent differential protein bands present in immuno-precipitated *35S::SPL13-GFP* alfalfa plants are indicated with black arrow in ‘a’. Abbreviations in ‘b’ represent: CHS, Chalcone synthase; DFR, dihydroflavonol-4-reductase; GDSL, Gly-Asp- Ser-Leu –lipase; GSK, Glycogen synthase kinase-3; IFR, Isoflavone reductase; PAL, Phenylalanine Ammonia-Lyase; PDH, Proline Dehydrogenase

IPMS was used to identify candidate proteins that interact with SPL13 in alfalfa under drought stress. In this experiment, alfalfa plants overexpressing *SPL13* tagged with a green fluorescent protein, *35S::SPL13-GFP* [17] and wild-type plants grown under control and drought stress conditions were used. Isolation of SPL13-interacting proteins followed by Coomasie staining showed the presence of unique protein bands in *35S::SPL13-GFP* plants that were lacking in wild-type (Fig. 6a**)**. There were some prominent and many faint bands. Therefore, to identify all the proteins present, a FASP proteomics method was employed on the total IP eluent. Subsequently, the precipitated proteins were digested with trypsin and identified by mass spectrometry. To account for the lack of a complete *M. sativa* proteome database, a combined use of contig database and partial Uniprot database identified unique candidate proteins that would have been missed by the *M. truncatula* database alone. We were able to identify candidate SPL13-interacting proteins involved in the photosynthesis process, specialized metabolite biosynthesis, ROS scavenging, and abiotic stress tolerance in addition to normal cellular activity-involved peptides (Table 1; Table S6).

**Table 1:** Detected candidate proteins interacting with SPL13 by IPMS analysis

Proteins	Reported roles and description	Sequence coverage (%)	# Peptides	# Spectra	Molecular weight (KDa)
50S ribosomal protein L22	Early stages of 50S ribosomal protein assembly	18	4	7	21.88
60s ribosomal protein L11	Biogenesis of 60S ribosomal subunits	49.72	6	11	20.67
Arginine biosynthesis bifunctional protein	Involved in the cyclic version of arginine biosynthesis	26.72	7	21	48.6
Beta-xylosidase/alpha-L-arabinofuranosidase	Releases xylose and arabinose from cell walls	25.32	15	30	83.67
Cafeic acid 3-O-methlyltransferase (COMT1)	Catalyzes the conversion of caffeic acid to ferulic acid and of 5-hydroxyferulic acid to sinapic acid.	17.81	6	31	39.92
Cell division control protein 2 homolog 1	control of the eukaryotic cell cycle	17.87	4	11	33.46
Chalcone synthase 1	naringenin-chalcone or chalcone biosynthesis	15.17	5	14	42.74
Chalcone synthase 2		31.62	9	38	42.68
Chalcone synthase 4		28.53	8	34	42.63
Cyclic beta-(1,2)-glucan synthase	biosynthesis of cyclic beta-(1,2)-glucan	15.11	29	86	315.55
Dihydroflavonol 4-reductase	anthocyanin biosynthesis	33.18	6	12	24.37
Eukaryotic translation initiation factor 5A-1	mRNA-binding protein involved in translation elongation	32.3	3	32	17.65
Glutamate synthasee [NADH] amyloplastic	assimilation of symbiotically fixed nitrogen into amino acids	19.51	30	99	240.22
Glutamine synthetase cytosolic isozyme	homeostatic control of glutamine synthesis	16.85	4	9	39.08
Glutamine synthetase leaf isozyme, chloroplastic	Re-assimilation of the ammonia generated by photorespiration	14.95	6	15	47.09
Glycogen synthase kinase-3 homolog − 1	Brassinosteroids signaling and abiotic stress response	21.41	6	11	46.74
Glycogen synthase kinase-3 homolog − 2		25.3	7	94	46.03
Glycogen synthase kinase-3 homolog − 3		37.23	11	27	46.24
Isoflavone reductase, IFR	Reduces achiral isoflavones to chiral isoflavanones	13.52	4	5	35.43
Long-distance movement protein	facilitate the cell-to-cell movement	17.93	3	3	26.67
Malate dehydrogenase, chloroplastic	Catalyzes a reversible NAD-dependent dehydrogenase reaction involved in central metabolism	16.87	3	3	35.52
Methylthioribose-1-phosphate isomerase	Catalyzes methylthioribose-1-phosphate (MTR-1-P) into methylthioribulose-1-phosphate (MTRu-1-P) interconversion	38.14	11	15	42.24
Mitogen-activated protein kinase homolog	mitogenic induction of symbiotic root nodules	21.19	6	32	44.37
Nodulation protein D1	Regulates other nodulation proteins	15.2	3	3	34.86
Nodulation protein E	synthesize NOD factor fatty acyl chain	26.68	7	9	41.64
Nodulin-25	development and function of nodules	19.51	3	6	27.51
Phenylalanine ammonia-lyase	catalyzes the deamination of phenylalanine to cinnamate and ammonia	21.38	12	21	78.82
Phosphoenolpyruvate carboxylase	catalyzes the addition of bicarbonate to phosphoenolpyruvate	17.08	11	16	110.82
Polygalacturonase	depolymerization of pectates	15.44	6	29	43.93
Replication protein 1A	stabilizes single-stranded DNA intermediates, that form during DNA replication or upon DNA stress.	33.13	25	170	111.95
Ribulose bisphosphate carboxylase large chain	Catalyzes the carboxylation of D-ribulose 1,5-bisphosphate and oxidative fragmentation of the pentose substrate	24.05	12	21	52.59
Ribulose bisphosphate carboxylase small chain	Catalyzes the carboxylation of D-ribulose 1,5-bisphosphate and oxidative fragmentation of the pentose substrate	46.67	7	34	20.24
RNA-directed RNA polymerase	catalyses synthesis of the RNA strand complementary to a given RNA template	15.14	9	34	93.6
RNA-directed RNA polymerase 2a	required for cytoplasmic and chromatin RNA silencing pathways	16.91	9	13	93.46
Serine/threonin-protein phosphatase PP1	essential for cell division and glycogen metabolism by dephosphorylating substrates	29.6	8	97	36.23
Serine/threonin-protein phosphatase PP2A	Dephosphorylates and activates the actin-depolymerizing factor ADF1	18.85	3	5	35.69
Sucrose synthase	Sucrose-cleaving enzyme that provides UDP-glucose and fructose	15.65	8	31	92.27
Tyrosine recombinase XerC	Catalyze the cutting and rejoining of the recombining DNA molecules	15.72	3	3	33.75
Vacuolar membrane protease	vacuolar sorting and osmoregulation	21.96	14	45	112.35
Vacuolar protein sorting/targeting protein 10	sorting receptor in the Golgi compartment required for the intracellular sorting and delivery of soluble vacuolar proteins	18.45	22	67	161.33

Candidate proteins were considered when at least three peptides with six spectra were present from the protein while undergoing the database search as specified in the method part of the article. Thermo Q-Exactive Orbitrap mass spectrometer coupled to an Easy-nLC 1000 nano LC system were used to analyze samples

### SPL13 interacts with photosynthesis- and phenylpropanoid pathway -related proteins

The identification of SPL13-interacting proteins indicated proteins involved in photosynthesis are among the top candidates. For example, there were multiple occurrences of both small and large chains of the ribulose bisphosphate carboxylase (Rubisco), photosynthesis I p700 chlorophyll a apoprotein A2, and phytochrome A (Table 1; Table S6).

A previous study reported that reduced binding of SPL13 to *DFR* promoter enhances anthocyanin biosynthesis [15]. Using the current IPMS analysis, we were able to identify DFR and other candidate proteins interacting with SPL13, such as phenylalanine ammonia-lyase1(PAL1) which catalyses the first step in the phenylpropanoid pathway by converting phenylalanine to cinnamate [37]. Other candidate peptides interacting with SPL13 from this pathway are chalcone synthase1, 2 and 4 (CHS1, CHS2, CHS4), which catalyzes the specialization from the general phenylpropanoid pathway into flavonoids converting 4-coumaroyl-CoA and malonyl-CoA [10]. Other specialized metabolite biosynthesis mediating proteins such as cafeic acid-3-o-methyltransferase (COMT1) and isoflavone reductase (IFR) were also found to interact with SPL13 (Table 1).

### SPL13 interacts with proteins involved in stress signal transduction and resilience

Perception of environmental changes followed by signal transduction are a prerequisite for orchestrating the necessary measures to alleviate the stress effects through various kinases. In our study, Glycogen synthase kinase-3 and Mitogen-activated protein kinase homologs were detected in *35S::SPL13-GFP* plants under drought stress (Table 1). Moreover, N+/H+ antiporter, which is involved in ion balance to mediate stress tolerance and cellular activity [58], was also detected by IPMS analysis as an interacting partner of SPL13 under drought stress (Table S6).

## Discussion

### Genotype-specific gene expression patterns in response to drought stress

Silencing of *SPL13*, a target of miR156, in alfalfa affected a plethora of genes leading to improved drought tolerance [2]. Among the commonly and significantly increased transcripts across the different tissues of *SPL13*RNAi alfalfa, *FLAP* was reported to have primarily a cell adhesion function and to play a role in abiotic stress tolerance in *Arabidopsis* [27, 60]. Moreover, *proline dehydrogenase* (*PDH*) was increased in *SPL13*RNAi. PDH is known for its role in drought tolerance by either scavenging ROS, balancing the carbon to nitrogen ratio through GABA shunt [18], or serving as an osmolyte [22]. Proline and its catabolic products, mediated through PDH, are more important to drought tolerance than the high accumulation of proline itself [7]. The other commonly increased transcripts with higher fold-changes were transcripts of ABA receptors known for their role in abiotic stress tolerance [32, 34]. Accordingly, the increased transcript levels of *FLAP, PDH,* and ABA receptors along with other transcripts across the three tissue types (leaf, stem and root) in *SPL13*RNAi alfalfa relative to EV suggests that these proteins play a major role in the response of *SPL13*RNAi plants to drought stress.

### Photosynthesis-related DEG are upregulated in leaves of *SPL13*RNAi plants during drought

Physiological investigation of *SPL13*RNAi plants under drought stress showed maintenance of vital physiological processes, such as photosynthesis and leaf water holding capacity [15]. This was accompanied by an upregulation of photosynthesis-related genes (*PSI* and *PSII*) in *SPL13*RNAi plants [15]. In the current study, comparison of transcript profiles of leaves of both genotypes under control and drought conditions revealed that various metabolic pathways were affected, with photosynthesis being predominantly higher in *SPL13*RNAi plants. The observed upregulation of photosynthesis-associated DEG was mainly in the light-dependent reaction, consistent with the physiological data report of the previous study [15] which showed that *SPL13*RNAi plants maintained the photosynthesis process during drought stress, while photosynthesis was reduced in EV plants. Other studies have shown that the light-dependent reaction centers were significantly affected by drought in maize [61, 63]. The maintained or slightly increased photorespiration-associated transcripts in *SPL13*RNAi plants may serve as an energy sink to prevent over-reduction of NADPH and photo inhibition in *SPL13*RNAi plants. Moreover, an increased level of photo-inhibition; unreduced NADPH with lower photosynthetic assimilation rate, was reported previously in drought-stressed barley plants [56]. A higher level of photosynthesis assimilation rate reported in the previous study [2, 15] along with the current transcript-based evidence in *SPL13*RNAi plants suggest a mechanism to potentially lower the reductive power of NADPH that could affect the photosynthesis otherwise.

### Specialized metabolism-related DEG are upregulated in stems of *SPL13*RNAi plants during drought

Specialized metabolites are important in plant growth and development, and their association with reduced ROS levels was reported in in vivo [1] and in vitro assays [41]. Accordingly, increasing the abundance of specialized metabolites and associated primary metabolites, such as ascorbates and proline, are considered as a marker for enhanced biotic and abiotic stress tolerance [24]. In our earlier study, we observed enhanced levels of primary and specialized metabolites related to stress tolerance, such as anthocyanin, in *miR156* overexpressing alfalfa genotypes [15]. This suggests anthocyanin and possibly other ROS scavenging metabolites are regulated by SPL13 in a miR156-dependent manner involving DFR. To further investigate the involvement of SPL13 in regulating levels of specialized metabolites, especially phenylpropanoids, the global transcript levels in leaf, stem and root tissues of *SPL13*RNAi plants was investigated relative to tissues from EV. Importantly, stem-derived samples showed an enhanced abundance of transcripts associated mainly with the phenylpropanoid pathway, unlike leaf and root tissues that showed an increase in mainly photosynthesis- and ion transport- associated transcripts, respectively. The enhanced abundance of transcripts associated with the phenylpropanoid pathway is consistent with the observed colour development in stems of *miR156* overexpressing plants [15]. An earlier study showed that the accumulation of anthocyanin was positively correlated with the level of *DFR* expression in potato in a non-tissue specific manner [54]. The ChIP-qPCR analysis revealed that while SPL13 indeed binds to *DFR* promoter presumably to regulate its expression [15], enhanced anthocyanin accumulation was observed only in the stem. RNAseq analysis also indicated upregulation of transcripts belonging to the biosynthesis of anthocyanin and other polyphenols from the phenylpropanoid pathway mainly in stem tissues during drought stress. Whether SPL13 also regulates the phenylpropanoid pathway in tissues other than the stems remains to be investigated further.

### The upregulated root-specific DEG are mainly attributed to ion transport in *SPL13*RNAi plants during drought stress

Roots are the first to encounter low moisture in the soil, but maintenance of plant water potential is not completely dependent on roots but rather on a continuum that involves soil, root, leaf, and the atmosphere via the transpiration stream [13, 36]. To maintain water potential, drought tolerant plants use different strategies to affect the osmotic balance and/or hydrostatic force governed by the transpiration stream. Osmolytes, such as sugars and proline, have been reported to adjust osmotic balance in different plants [24, 45]. Previously, we showed drought tolerant genotypes of *miR156*OE had higher levels of the osmolytes proline and sugars [2, 15]. To understand the involvement of SPL13 in maintaining water potential under drought, transcripts from *SPL13*RNAi and EV root tissues were profiled under control and drought conditions. The DEG showed increased levels of transcripts associated with GABA shunt and membrane integrity, such as *GDSL*, in *SPL13*RNAi plants. Primary metabolites, such as ascorbate and glutathione, and phenylpropanoid specialized metabolites known to scavenge ROS were also significantly increased. To validate the transcript-based metabolite pathway analysis and identify non-enzymatic metabolite conversions [28], primary and specialized metabolite analysis in *SPL13*RNAi plants is important. The eventual release of the alfalfa genome sequence should allow for pathway analysis to potentially identify novel metabolite pathways unmapped to the *M. truncatula* genome in the current study, but which may contribute to drought stress response in alfalfa.

### SPL13 interacts with proteins involved in photosynthesis, specialized metabolite biosynthesis, stress signalling and ROS scavenging processes

Previously, we showed that silencing of *SPL13* in alfalfa led to the maintenance of photosynthesis under drought stress by affecting genes involved in this process [15]. In the current study, an enhanced level of transcripts associated with photosynthesis, mainly light reaction, was observed in leaf tissues. Consistent with this finding, IPMS analysis showed that SPL13 interacts with photosynthesis- related proteins such as RUBISCO (small and large chain) and PSI. Rubisco is composed of eight small sub units encoded by the ribulose-1,5-bisphosphate carboxylase/oxygenase small subunit (*RBCS*) and eight large subunits encoded by *RBCL* [50]. Posttranslational modification of both the small and large subunits of RUBISCO is required for functional activity [25] and functional-holoenzyme assembly [12]. Accordingly, modifications to either subunits of RUBSICO result in reduced photosynthetic activity and retarded growth [46], and any interactions these subunits have may influence the enzyme activity. In this regard, the increased binding of the small and large chains of RUBISCO to SPL13 under drought stress in *SPL13* overexpressing genotypes may interfere with the subunits assembly. Furthermore, alfalfa plants with reduced level of *SPL13* (*SPL13*RNAi plants) were previously reported to have a higher level of maximum rate of rubisco carboxylase activity (*V*_cmax_) under drought stress [15]. Investigating SPL13 binding sites in both small and large chain subunits of RUBISCO would provide knowledge on how the miR156/SPL13 module maintains photosynthesis efficiency under drought stress.

PAL, DFR and CHSs were among the protein candidates identified that interact with SPL13 as detected by IPMS in the current study. These enzymes mediate the biosynthesis of specialized metabolites in the phenylpropanoid pathway, and alteration of their expression levels affects abundance of these metabolites in plants. For examples, Arabidopsis plants with reduced expression of PAL (PAL1 to PAL4) had reduced content of proanthocyandins in the seed coats, and developed ultraviolet light sensitivity [26]. Likewise, enhanced levels of CHS, which mediates the step-wise condensation of malonyl-CoA with coumaroyl-CoA to form naringenin chalcone (flavonoids), was associated with increased accumulation of anthocyanin abundance and stress tolerance in plants [11, 48]. The regulation of PAL, DFR, and CHSs, may explain the enhanced biosynthesis of phenolic compounds needed to scavenge ROS in *miR156*OE and *SPL13*RNAi alfalfa plants [15].

Plants use various stress signal transduction mechanisms, which among others involve mitogen-activated protein kinase and mitogen-activated protein kinase kinase [16, 20, 43]. In the current study, we detected the Glycogen synthase kinase-3 homologs (GSK3_1, 2,and 3) along with one of its phosphorylated target Serine/threonin-protein phosphatase (PP1 and PP2A) as an interacting partner of SPL13. Brassinosteroid-involving GSK3s are involved in cell signalling [9, 47, 59] and regulate mitogen-activated protein kinase during abiotic stress response for stomatal regulation [29, 30]. The IPMS data showed that the above stress signal transduction pathway and its regulators interact with SPL13, suggesting a strategy used by alfalfa plants to respond to drought.

## Conclusions

Based on previous reports on the role of miR156/SPL13 module in regulating drought response in alfalfa [15], we investigated whether the role of miR156/SPL13 in drought response is tissue-specific. In this study, the global transcriptomic profiles of *SPL13*RNAi plants showed tissue-specific regulation of transcripts and associated pathways. In leaf tissues, there was an increase in the transcript levels of mainly photosynthesis- and photorespiration-related genes in *SPL13*RNAi plants. On the other hand, the stem tissues of *SPL13*RNAi plants exposed to drought showed an increase in transcripts related to the phenylpropanoid pathway. Furthermore, roots of *SPL13*RNAi plants under drought had an increase in transcripts associated with ion transporters, as well as primary and specialized metabolism, presumably to transport osmolytes and scavenge ROS while maintaining membrane integrity through GDSL. Moreover, SPL13 was found to interact with proteins involved in photosynthesis process, stress tolerance, and specialized metabolite biosynthesis.

We propose a model for a drought tolerance mechanism in which moderate levels of *miR156* overexpression regulate SPL13 in a tissue-dependent manner (Fig. 6b). *miR156* is induced by drought stress, which in turn silences *SPL13* [2]. Reduced expression of *SPL13* driven by *miR156* and enhanced level of WD40–1 enhances *DFR* [15], resulting in the accumulation of anthocyanins mainly in stem tissues. Similarly, silencing of *SPL13* would allow for continued activity of GSK3 signalling along with PAL, CHS, and DFR due to reduced interaction of these proteins with SPL13, further fueling the biosynthesis of drought stress-alleviating specialized metabolites, and facilitating ROS scavenging in *SPL13*RNAi and *miR156*OE alfalfa plants under drought stress. Future structural and enzymatic investigation of the candidate SPL13-interacting proteins could elucidate the active sites of the proteins, and their modifications needed to regulate drought stress in alfalfa. It is also important to validate the protein-protein interactions detected in this study using other techniques, such as yeast-two-hybrid assay.

## Methods

### Plant material

#### *SPL13* overexpressing and *SPL13*RNAi alfalfa plants

Alfalfa (*Medicago sativa* L.) genotypes with reduced expression levels of *SPL13*, *SPL13*RNAi-6 genotypes [2], empty vector control (EV) [5], *35S::SPL13-GFP* [17], and wild-type (WT) were used in this study. The transgenic alfalfa plants were generated previously in Dr. Hannoufa’s laboratory using the WT clone N4.4.2 [6] that was obtained from Dr. Daniel Brown (Agriculture and Agri-Food Canada). The transgenic alfalfa plants are not deposited in a public herbarium, so requests for any plant material should be directed to the corresponding author. The plants were grown in a fully automated greenhouse with 16-h light (380–450 W/m^2^), relative humidity (RH) of 70% and temperature of 25 ± 2 °C at the Agriculture and Agri-Food Canada London Research and Development Center, London, Ontario, Canada. Given that alfalfa is an obligatory outcross, we used vegetative cuttings for propagation according to Aung et al. [5] to maintain genotypes throughout the study.

#### Imposing drought stress

Drought stress was imposed on alfalfa plants devoid of water for 2 weeks at 30 days post vegetative propagation (juvenile vegetative stage) during which time plants were kept in a completely randomized design. Equal soil moisture levels were maintained before starting the experiment using a SM 100 soil moisture sensor (Spectrum Technologies Inc., Jakarta, Indonesia). Three biological replicates were used per genotype per treatment for transcript analysis (each replicate being an individual plant). Leaves (newly developed upper leaves), stems (lower 5 cm internode close to soil) and roots (7.5 cm of main and auxiliary root tips) were harvested from *SPL13*RNAi and EV plants for total mRNA extraction. Samples were flash frozen with liquid nitrogen and kept at − 80 °C for later transcriptomic analyses.

#### RNA extraction

Lower basal stem internode, young top leaves and root tip samples were collected and flash frozen in liquid nitrogen and kept in a -80 °C freezer until used for qRT-PCR analysis and RNA sequencing. Approximately 50 mg fresh weight was used for total RNA extraction using a PowerPlant® RNA isolation kit (Cat # 13500) for leaf samples, a QIAGEN RNeasy® Plant mini kit for stem and root tissues (Cat # 74904), and a PowerLyzer®24 bench top bead-based homogenizer (Cat # 13155) following manufacturers protocols. Total RNA quality was verified using BioRad Bioanalyzer for integrity and Nanodrop concentration before RNAseq analysis.

#### RNAseq analysis

mRNA stranded library preparation followed by Illumina HiSeq 2500 RNA-sequencing with pair end of 126 nucleotide bases were performed as a fee-for-service at The Center for Applied Genomics, The Hospital for Sick children, Toronto, ON, Canada. RNAseq data was analyzed according to Trapnell et al. [52] on biocluster with shell scripts of Linux. The scripts used for the analysis are provided as supplementary file (Table S7). To identify expression pattern of genes and module identification, R-software (V.4.0) environment-based network analysis with weighted gene co-expression network, WGCNA, in ‘BiocManager’ package was performed according to Langfelder and Horvath [31]. Using the ‘featureCounts’ [33], the exon read counts were obtained from ‘.bam’ extension files of RNA sequenced samples followed by Principal Component Analysis in R-software.

Visualization of differentially expressed genes-based pathway analysis was carried out using MapMan free software V3.6 (https://mapman.gabipd.org/) with a *M. truncatula* reference genome sequence, Mt4.0 V2 (http://www.medicagogenome.org/downloads). Using an online free gene ontology analysis tool (http://revigo.irb.hr/), lists of differentially expressed genes with significance *p* values between genotypes and tissue types, the corresponding tree map codes for molecular function, biological process and cellular components were exported. Subsequently, the codes for generating the tree maps were visualized using R-software. Raw RNA sequencing reads can be accessed at the National Center for Biotechnology Information, NCBI, BioProject PRJNA598830.

#### qRT-PCR analysis to validate RNAseq results

To validate the results from RNAseq analysis, the extracted RNA was treated with Ambion®TURBO DNA-*free™* DNase (Cat # AM1907) followed by iScript™ cDNA synthesis (Cat # 1708891). Medtr3g498825: *basic helix-loop-helix 137 bHLH137* (F:- CAGGATAATGCAGCAGAAGG, R:- GGGCTCATCCAAACACTTTC); Medtr7g109510: *basic leucine zipper bZIP* (F:- AGTTCGGGTTCTGATGGTGT, R:- CTCCAACAGTTTCTGCTTCC); Medtr5g048850: *flavonol synthase/flavanone 3-hydroxylase F3H* (F:- TTGTTCTAGGTGTGCCTCCA, R:- GAATGACAAGGGCATTAGGG); Medtr3g091350: *flavonol synthase/flavanone 3-hydroxylase F3H-2* (F:- TCTCTCCTGGTTCCTTCTGT, R:- GGTCGATAACAGGAACTTGT); Medtr5g081860: *MYB transcription factor MYB51* (F:- GATGGCTTAAGGTTGCTGAG, R:- GATAGCCAGGAATCGGAACA); Medtr5g090620: *UDP-glucose flavonoid 3-O-glucosyltransferase UDPFG* (F:- CGTGATAACCGTCTCCGAAT, R:- TGATGACCTGGAGAGAATCG); Medtr7g071050: *UDP-glucosyltransferase family protein UDPGT* (F:- CTTCTGGAAAGGCAAGATGG, R:- GCTCCTTCTTTGGTTGTTGG) genes were used for validation. The gene primers were designed using publicly available Primer3 software (http://primer3.ut.ee/) based on *M. truncatula* genome sequence and amplified product was sequenced for an identity check. Primer efficiency was checked before proceeding using for qRT-PCR analysis. qRT-PCR was performed using the CFX96™ Real-Time PCR detection system and SsoFast™ EvaGreen® Supermixes (Bio-Rad Cat # 1725204). Specifically, 2 μL cDNA (equivalent to 200 ng cDNA), 1 μL forward and reverse gene-specific primers (10 μM each), 5 μL SsoFast Eva green Supermix, and 2 μL of nuclease-free water was used to make the final reaction volume of 10 μL. PCR amplification was performed at: cDNA denaturation at 95 °C for 30 s followed by 40 cycles of 95 °C for 10 s, 58 °C for 30 s and 72 °C for 30 s (denaturation, annealing and extension, respectively) followed by a melting curve that runs from 65 °C to 95 °C with a gradual increment of 0.5 per 5 s. All reactions were performed with three technical replicates. Transcript levels were analysed relative to *acetyl-CoA carboxylase1* (F*:-* GATCAGTGAACTTCGCAAAGTAC*,* R:- CAACGACGTGAACACTACAAC) and *ACTIN* (F:- TCAATGTGCCTGCCATGTATGT*,* R:- ACTCACACCGTCACCAGAATCC) housekeeping genes designed based on alfalfa sequence [15]. The selected differentially expressed genes from the RNAseq result were subjected to fold-change in different tissues of *SPL13*RNAi-6 and EV plants and compared to qRT-PCR results as described in Wang et al., [55]. The values are presented with regression analysis (Fig. S5**)** indicating a positive correlation (*R*^2^ = 0.74) with some differences associated with sensitivity of the two platforms.

#### Isolation of SPL13-interacting proteins

To determine SPL13-interacting proteins, we used alfalfa plants expressing SPL13 tagged with GFP proteins (*35S::SPL13-GFP*) and wild-type alfalfa plants. Plants were subjected to drought stress for 2 weeks prior to protein extraction. Subsequently, samples were immediately flash frozen using liquid nitrogen and kept in − 80 °C until use. μMACS GFP Isolation Kit (product number 130–091-125) was used to separate SPL13 interacting proteins using the manufacturer’s instructions (Miltenyi Biotec, USA). In brief, plant tissue was ground using a mortar and a pestle in liquid nitrogen and 1 g tissue was used for analysis. Two ml of pre-cooled lysis buffer was added along with 50 μL proteinase inhibitor cocktail and kept on ice for 30 min with occasional mixing. The samples were centrifuged at 10000 g for 10 min and 50 μL of anti-GFP tag microbeads was added to the supernatant to magnetically label the epitope-tagged SPL13, mixed and kept on ice for 30 min. A total of 50 μL of the extract was kept as input control while the rest of the sample was eluted on μMCAs separator. After four rounds of stringent wash to remove non-interacting proteins with 200 μL of wash buffer 1, a final 100 μL wash buffer was used to remove the remaining unbound proteins and the eluent was used as the negative control. Finally, 20 μL of elution buffer (heated at 95 °C) was added to the column, and the column was incubated for 5 min before adding the final 50 μL of heated elution buffer and recovering the eluent.

#### SDS-PAGE electrophoresis

Proteins were resolved using SDS-polyacrylamide gel electrophoresis (PAGE). Samples were heated at 80 °C for 5 min to better resolve proteins. The electrophoresis was set for 30 min with 110 V followed by 125 v until the loading dye reached the bottom of the gel (~ 1.5 h) using vertical Mini-PROTEAN® Tetra cell system (BioRad, Catalog # 1658000, Mississauga, Canada). Gels were washed twice with distilled water and treated with Coomasie solution (Acetone: Methanol: distilled water 10:45:45 with 0.25% Brilliant Blue G-250), de stained with de-staining solution (Acetone: Methanol: distilled water 10:45:45) until protein bands were observed. The protein gels were then visualized using Gel DocTM EZ imager system (BioRad, Catalog # 1708270).

#### FASP – LC-MSMS-based protein identification

Proteins pulled down by immunoprecipitation were identified by LC-MS/MS with filter aided sample preparation (FASP), according to Wiśniewski et al., [57] with modifications. Briefly, 50 μl of the proteins eluted in the IP step were loaded onto a 10 kDa size exclusion Amicon® pro filter (Millipore Sigma Catalog # ACS501024). The solvent was exchanged with 150 μl of 8 M urea (Sigma, U5128), incubated at 40 °C for 25 min followed by centrifugation (14,000 G, 15 min). The elute was reduced with 100 mM DTT in Urea and alkylated with 100 μl of 50 mM iodoacetamide in a dark for 20 min. The proteins were washed twice with 100 μl of 50 mM ammonium bicarbonate in water and digested with 2 μg trypsin overnight at 34 °C. The digested peptides were then pulled through the molecular weight cut-off filter and recovered by washing with 1 ml 0.1% formic acid in LC-MS grade water. Solid-Phase Extraction (SPE) was performed using Waters OASIS HLB cartridge (Waters, SKU: 186003365) in a vacuum chamber. Subsequently, peptides were dried on a centrivap and reconstituted in 100 μL acetonitrile:water (95:5) with 0.1% formic acid and analyzed by a Thermo Q-Exactive Orbitrap mass spectrometer coupled to an Easy-nLC 1000 nanoLC system according to Fan et al. [14].

#### Database development and peptide identification for IPMS

To identify peptides in alfalfa, previous reports used either the *Medicago truncatula* proteome database [8, 62] or combinations of green plants (viridiplantae) [40, 44], despite relatively low identity matches. In the current study, we took a different approach by constructing a new peptide database derived from alfalfa transcriptomic contigs, followed by confirmation with a partial (2201 amino acid sequences) *M. sativa* database and *M. truncatula* protein database. Due to the absence of information on where the contig sequences are located relative to the genes transcription start sites (TSS), first, we translated 112,626 alfalfa transcript contigs followed by two additional frameshifts. When the translation process reached an early stop codon, the following sequences were assigned to the same contig but with a unique extension number (e.g. contig_1, contig_1_2). Moreover, any contig, or its extension, containing less than five amino acids were removed from the database. Subsequently, we were able to generate a 5,859,496 alfalfa peptide sequence database, from now onwards referred to as ‘contig database’ (Table S5). Peptide identity search from the samples was performed using the contig database and candidates containing at least three peptides and six spectra were considered for further analysis. The top SPL13-interacting candidate peptides were functionally annotated using the *M. truncatula* and *M. sativa* protein databases, by best fit. To confirm the results, peptide identification was repeated with the candidate peptide contigs replaced by *M. truncatula* homolog sequences in the contig database. Additionally, as of January 8, 2020, there are 2201 *M. sativa* protein sequences on the Uniprot protein database (https://www.uniprot.org/uniprot/) derived from manual annotation (140) and computational analysis (2061). Hence, we used the Uniprot database to determine whether the contig database-identified peptides are detectable with this analysis, and also to search for other SPL13-interacting proteins.

A fixed modification of carbamidomethylation (57.02) and a variable Oxidation modifications (15.99) of the target compounds were considered while undertaking the database search. Moreover, other protease and fragmentation parameters: such as trypsin as a cleavage enzyme; two maximum missed cleavages; precursor m/z tolerance of 12.0 ppm; fragment m/z tolerance of 0.08 Da, precursor charge of 2–4 with one isotope level were incorporated while searching and identifying for the candidate proteins. Peak lists obtained from MS/MS spectra were identified using X!Tandem version X! Tandem Vengeance (2015.12.15.2) [PMID 14976030], Andromeda version 1.5.3.4 [PMID 21254760] and MyriMatch version 2.2.140 [PMID 17269722]. The peptide search was conducted using SearchGUI version 3.3.20 [PMID 21337703] and were inferred from the spectrum identification results using PeptideShaker version 1.16.45 [PMID 25574629] [53].

### Statistical analysis

All statistical data analyses were undertaken using R-software environment 4.0. Shell scripts of Linux and R-software scripts used to analyze and visualize, respectively, the RNA sequence reads are provided as a supplementary file (Table S7).

## Supplementary information

**Additional file 1: Fig. S1.** Visualization of total exon read counts and library sizes generated from each biological samples. (**a**) Exon read counts library sizes, (**b**) log2 transformed exon read counts for constructing PCA plots, (**c**) Weighted Gene Co-expression Network Analysis (WGCNA) –based transcript analysis to visualize co-expression trend between genotypes and among tissues. **Fig. S2** Visualization of differentially expressed genes-associated pathways between drought-stressed leaf tissues of SPL13RNA and EV plants. (**a**) Molecular function tree map, (**b**) Biological process tree map, (**c**) Cellular component tree map. The free online gene ontology analysis tool (http://revigo.irb.hr/) was used to generate codes to construct tree map in R-software. **Fig. S3** Visualization of differentially expressed genes-associated pathways between drought-stressed stem tissues of SPL13RNA and EV plants. (**a**) Molecular function tree map, (**b**) Biological process tree map, (**c**) Cellular component tree map. The free online gene ontology analysis tool (http://revigo.irb.hr/) was used to generate codes to construct tree map in R-software. **Fig. S4** Visualization of differentially expressed genes-associated pathways between drought-stressed root tissues of SPL13RNA and EV plants. (**a**) Molecular function tree map, (**b**) Biological process tree map, (**c**) Cellular component tree map. The free online gene ontology analysis tool (http://revigo.irb.hr/) was used to generate codes to construct tree map in R-software. **Fig. S5** Validation of selected differentially expressed genes using quantitative real-time PCR (qRT-PCR) in different tissues of *SPL13*RNAi-6 and EV under drought conditions. Although an observed fold-change value differences between RNAseq data and qRT-PCR a linear regression (*R*^2^ = 0.74) indicates a positive correlation between the two platforms but reflecting a different sensitivity. Genes of Medtr3g498825: *basic helix-loop-helix 137 bHLH137*; Medtr7g109510: *basic leucine zipper bZIP*; Medtr5g048850: *flavonol synthase/flavanone 3-hydroxylase F3H*; Medtr3g091350: *flavonol synthase/flavanone 3-hydroxylase F3H-2*; Medtr5g081860: *MYB transcription factor MYB51*; Medtr5g090620: *UDP-glucose flavonoid 3-O-glucosyltransferase UDPFG*; Medtr7g071050: *UDP-glucosyltransferase family protein UDPGT* were used for comparisons while *acetyl-CoA carboxylase1* and *ACTIN* were used as reference genes. (PPTX 1486 kb)

**Additional file 2: Table S1.** List of genes differentially expressed between drought stressed *SPL13*RNAi and EV plants. DEG that are (**a**) increased and (**b**) decreased in drought-stressed *SPL13*RNAi plants compared to drought stressed EV plants. The differentially expressed genes are categorized as common to all tissues followed by tissue-specific (leaf, stem, or root tissues) expressions. *N* = 3 biological samples for each condition, where biological samples represent individual plants treated with similar treatment exposure.

**Additional file 3: Table S2.** List of genes differentially expressed between control and drought stressed *SPL13*RNAi plants**.** DEG that are (**a**) increased and (**b**) decreased in drought-stressed *SPL13*RNAi plants. The differentially expressed genes are categorized as common to all tissues followed by tissue-specific (leaf, stem, or root tissues) expressions. N = 3 biological samples for each condition, where biological samples represent individual plants treated with similar treatment exposure.

**Additional file 4: Table S3.** List of genes differentially expressed between control and drought stressed EV plants. DEG that are (a) increased and (b) decreased in drought-stressed EV plants. The differentially expressed genes are categorized as common to all tissues followed by tissue-specific (leaf, stem, or root tissues) expressions. N = 3 biological samples for each condition, where biological samples represent individual plants treated with similar treatment exposure.

**Additional file 5: Table S4.1** Full list of components included in molecular function, biological process and cellular component in leaf tissues**Table S4.2** Full list of components included in molecular function, biological process and cellular component in stem tissues**Table S4.3** Full list of components included in molecular function, biological process and cellular component in root tissues

**Additional file 6: Table S5** *Medicago sativa* contig database (FASTA 237506 kb)

**Additional file 7: Table S6** List of candidate peptides interacting with SPL13

**Additional file 8: Table S7.**. Shell scripts of Linux and R-software scripts used to analyze and visualize data.

## Data Availability

Raw RNA sequencing reads can be accessed at the National Center for Biotechnology Information, NCBI, BioProject PRJNA598830.

## Abbreviations

ChIP: Chromatin Immunoprecipitation
CHS: Chalcone synthase
COMT: Cafeic acid-3-O-methyltransferase
DEG: Differentially expressed genes
DFR: dihydroflavonol-4-reductase
EV: Empty Vector construct
FASP: Filter-assisted proteomics
FLAP: Fasciclin-Like Arabinogalactan Protein
GABA: Gamma aminobutyric acid
GDSL: Gly-Asp- Ser-Leu –lipase
GO: Gene Ontology
GSK: Glycogen synthase kinase-3
IFR: Isoflavone reductase
IPMS: ImmunoPrecipitation-based Mass Spectrometry
miR156: microRNA156
NCBI: National Center for Biotechnology Information
PAGE: Polyacrylamide gel electrophoresis
PAL: Phenylalanine Ammonia-Lyase
PCA: Principal Component Analysis
PDH: Proline Dehydrogenase
PP1: Serine/threonin-protein phosphatase
ROS: Reactive Oxygen Species
RUBISCO: Ribulose-1,5-bisphosphate carboxylase/oxygenase
SPE: Solid-Phase Extraction
TSS: Transcription Start Sites
WGCNA: Weighted Gene Co-expression Network Analysis
