# Supplementary material for: Transcriptome-IPMS analysis reveals a tissue-dependent miR156/SPL13 regulatory mechanism in alfalfa drought tolerance
Source: BMC Genomics. 2020 Oct 19;21:721. doi: 10.1186/s12864-020-07118-4 (PMC7574311; doi:10.1186/s12864-020-07118-4)
Supplement: Supplementary file 1 — Additional file 1: Fig. S1. Visualization of total exon read counts and library sizes generated from each biological samples. (a) Exon read counts library sizes, (b) log2 transformed exon read counts for constructing PCA plots, (c) Weighted Gene Co-expression Network Analysis (WGCNA) –based transcript analysis to visualize co-expression trend between genotypes and among tissues. Fig. S2 Visualization of differentially expressed genes-associated pathways between drought-stressed leaf tissues of SPL13RNA and EV plants. (a) Molecular function tree map, (b) Biological process tree map, (c) Cellular component tree map. The free online gene ontology analysis tool (http://revigo.irb.hr/) was used to generate codes to construct tree map in R-software. Fig. S3 Visualization of differentially expressed genes-associated pathways between drought-stressed stem tissues of SPL13RNA and EV plants. (a) Molecular function tree map, (b) Biological process tree map, (c) Cellular component tree map. The free online gene ontology analysis tool (http://revigo.irb.hr/) was used to generate codes to construct tree map in R-software. Fig. S4 Visualization of differentially expressed genes-associated pathways between drought-stressed root tissues of SPL13RNA and EV plants. (a) Molecular function tree map, (b) Biological process tree map, (c) Cellular component tree map. The free online gene ontology analysis tool (http://revigo.irb.hr/) was used to generate codes to construct tree map in R-software. Fig. S5 Validation of selected differentially expressed genes using quantitative real-time PCR (qRT-PCR) in different tissues of SPL13RNAi-6 and EV under drought conditions. Although an observed fold-change value differences between RNAseq data and qRT-PCR a linear regression (R2 = 0.74) indicates a positive correlation between the two platforms but reflecting a different sensitivity. Genes of Medtr3g498825: basic helix-loop-helix 137 bHLH137; Medtr7g109510: basic leucine zipper bZIP; Medtr5g04885 [file 12864_2020_7118_MOESM1_ESM.pptx]

## Slide 1
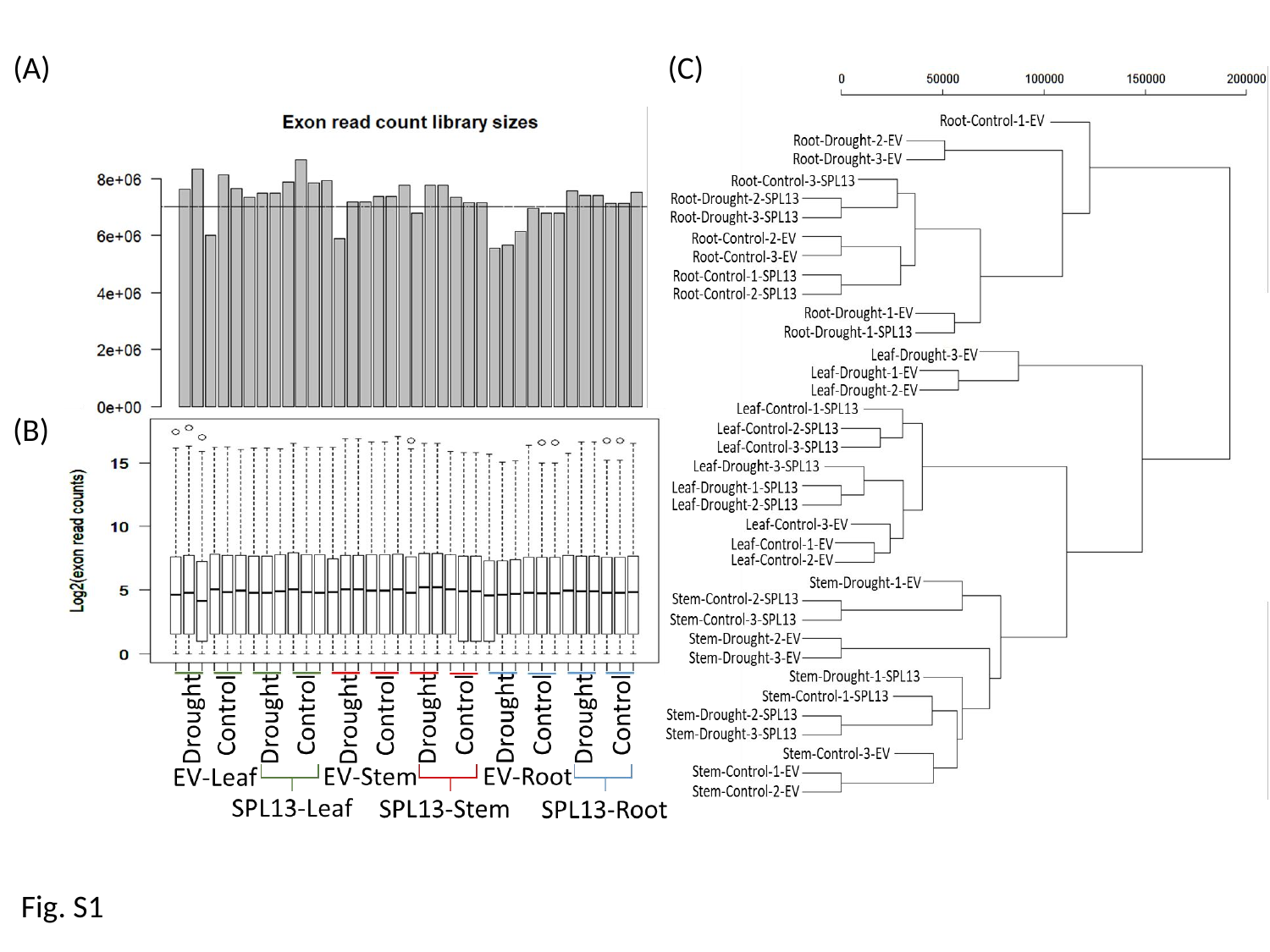

(A)
(C)
(B)
Fig. S1

## Slide 2
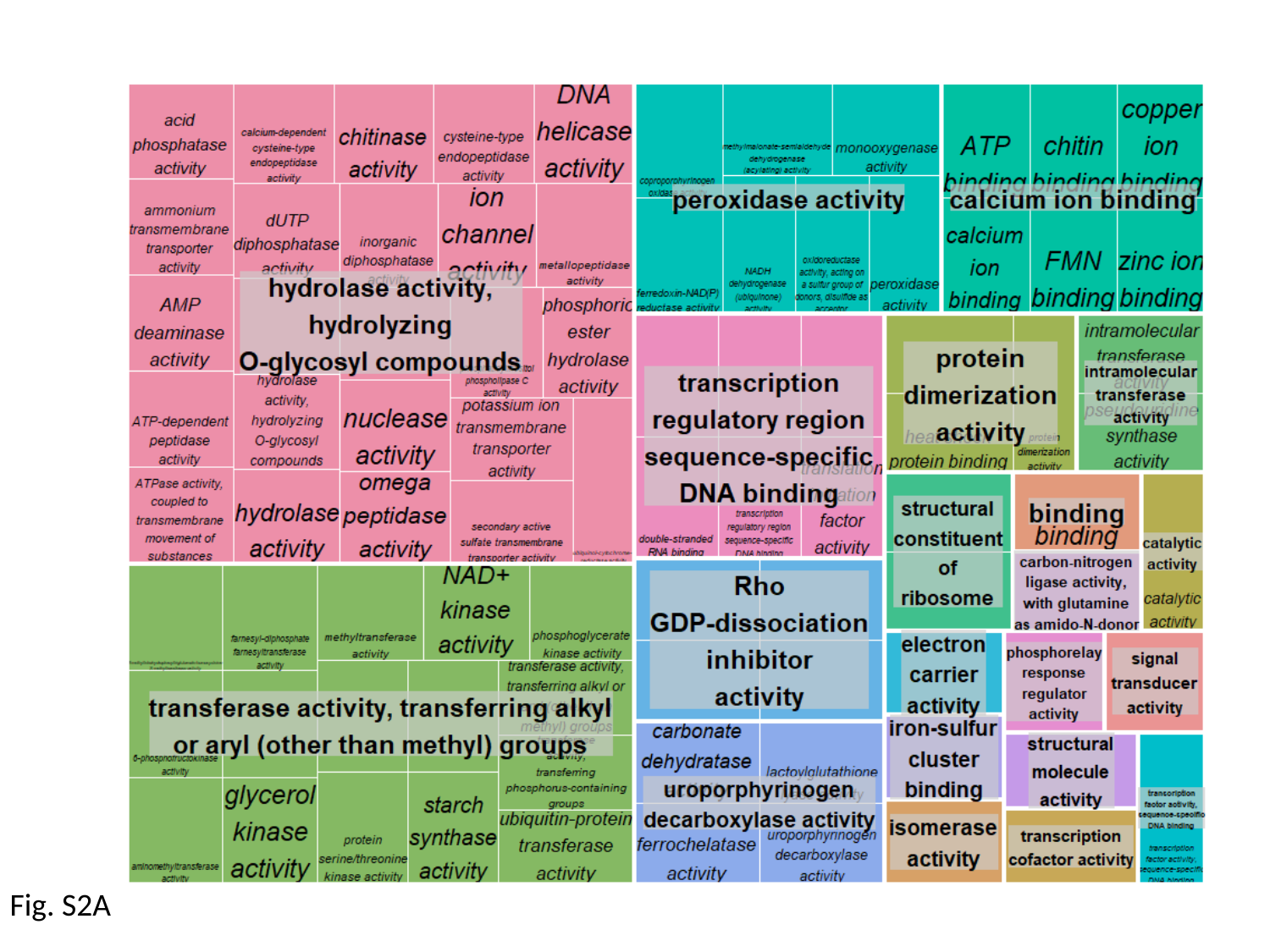

Fig. S2A

## Slide 3
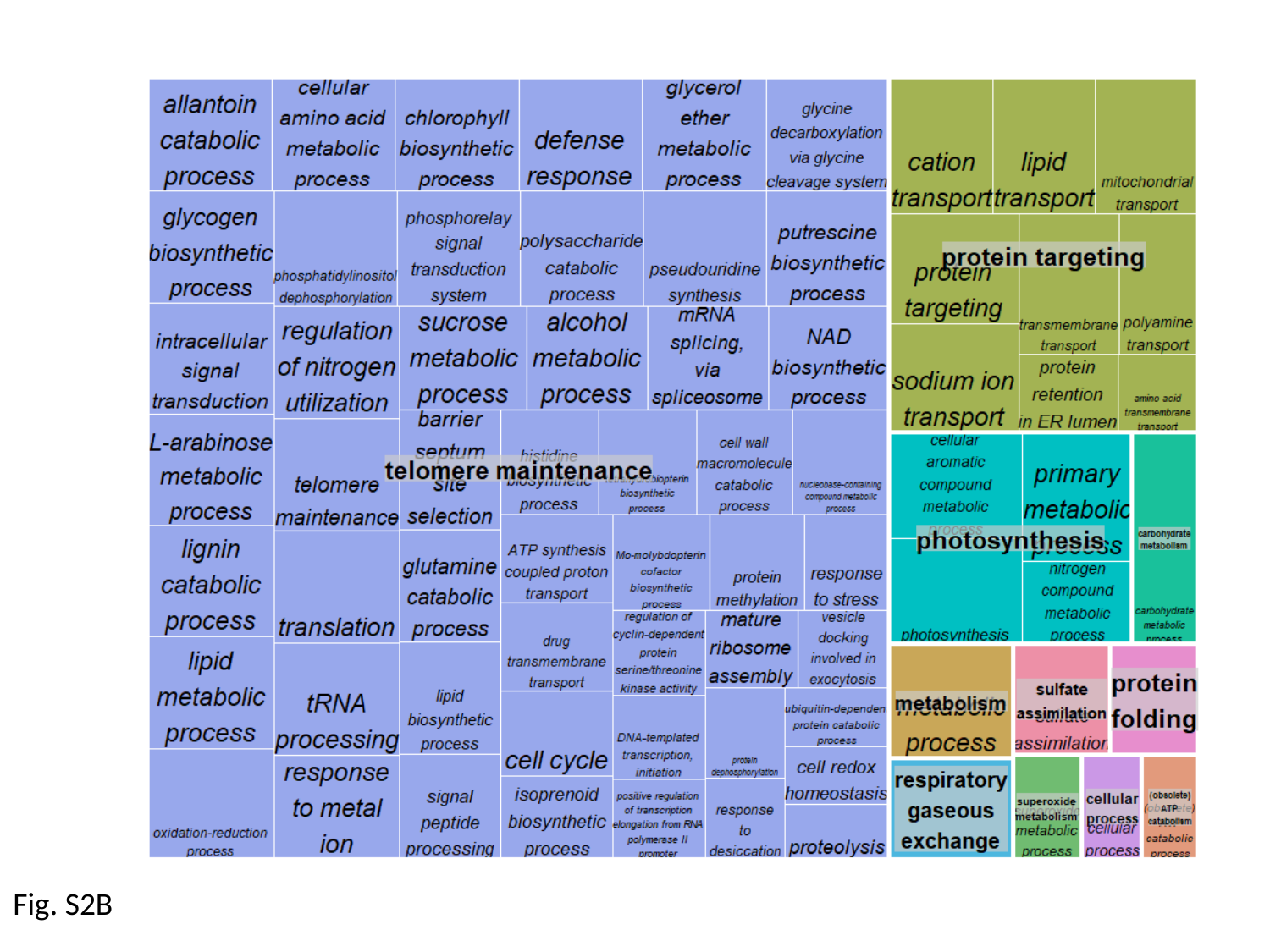

Fig. S2B

## Slide 4
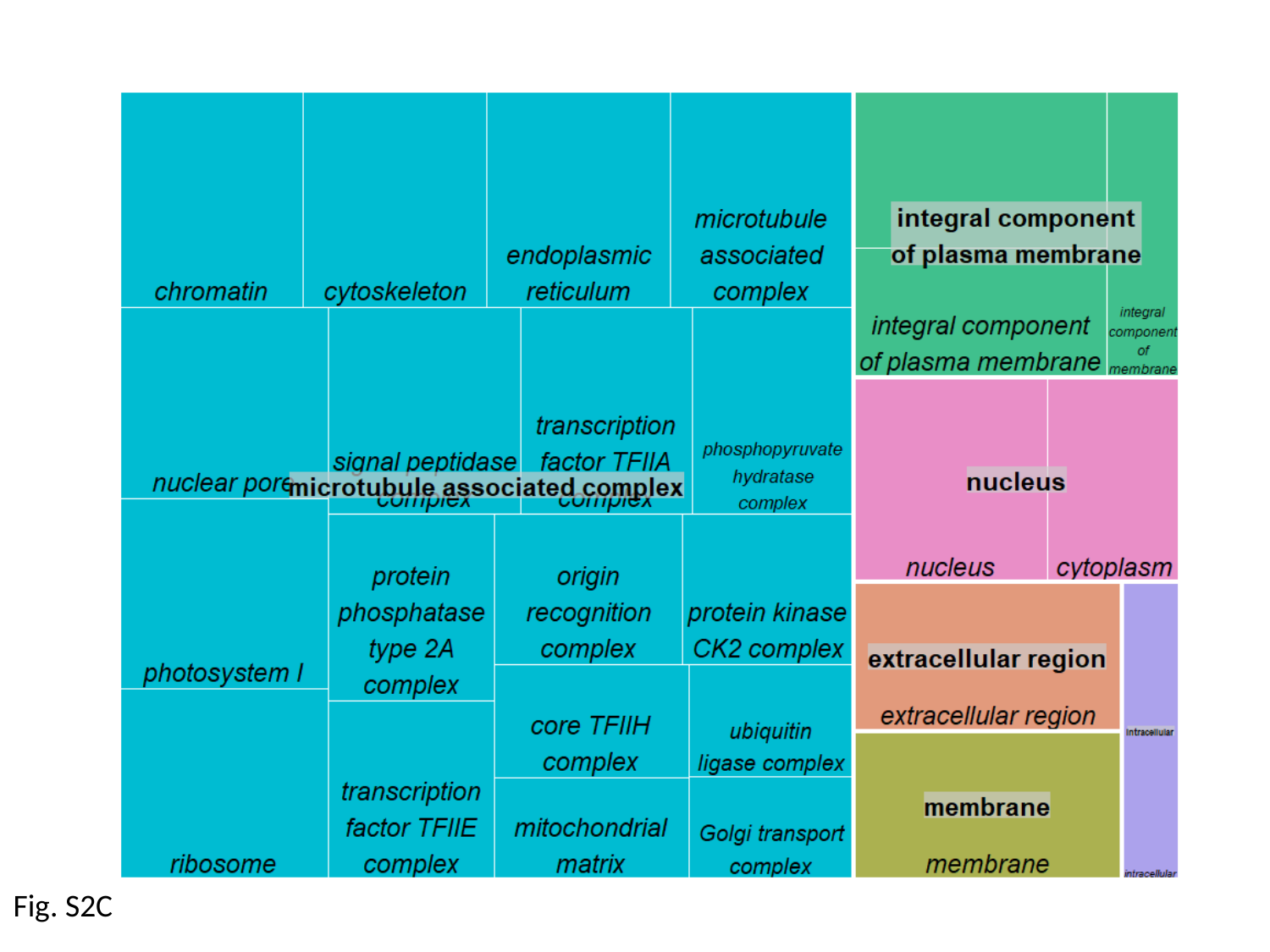

Fig. S2C

## Slide 5
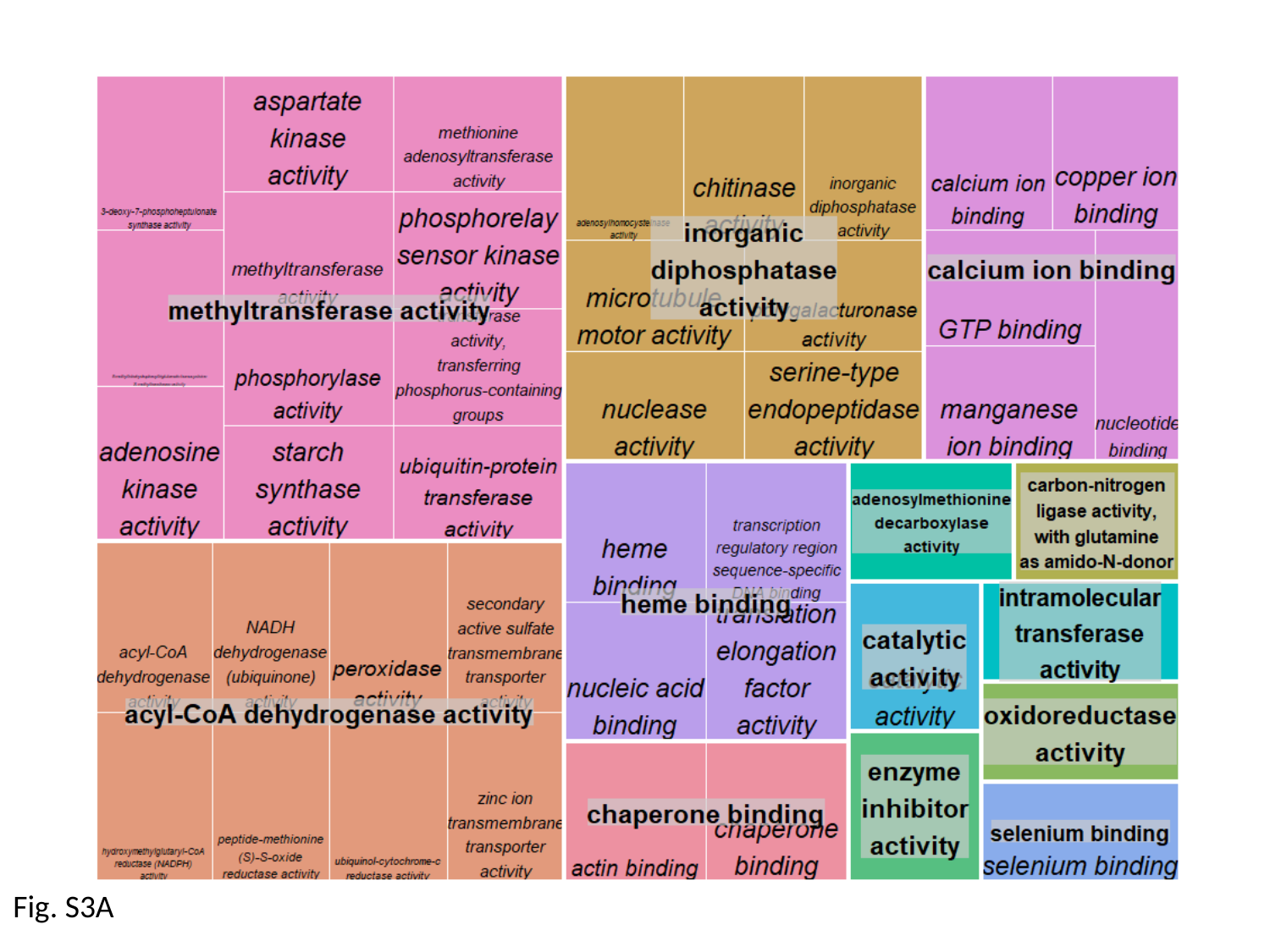

Fig. S3A

## Slide 6
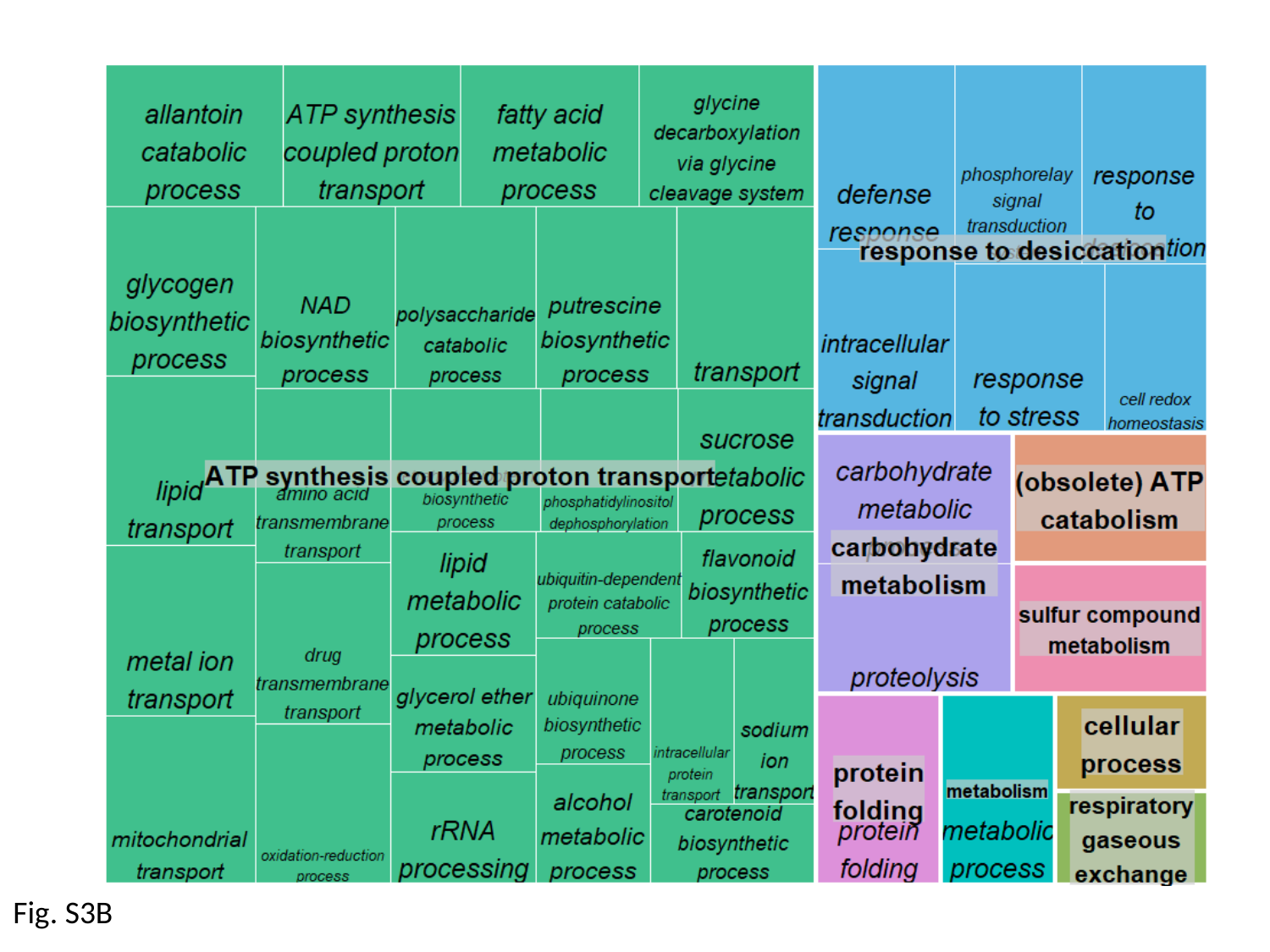

Fig. S3B

## Slide 7
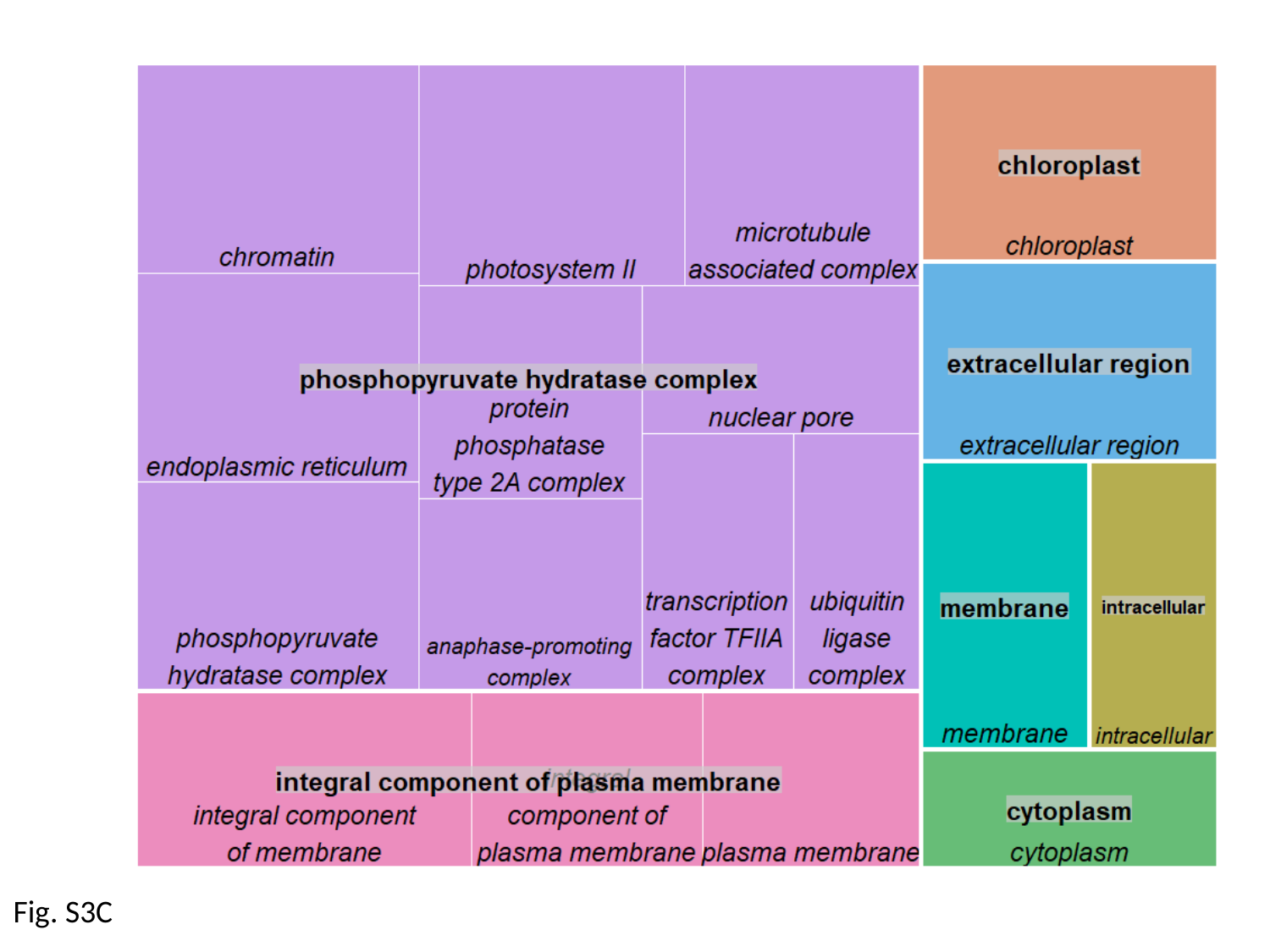

Fig. S3C

## Slide 8
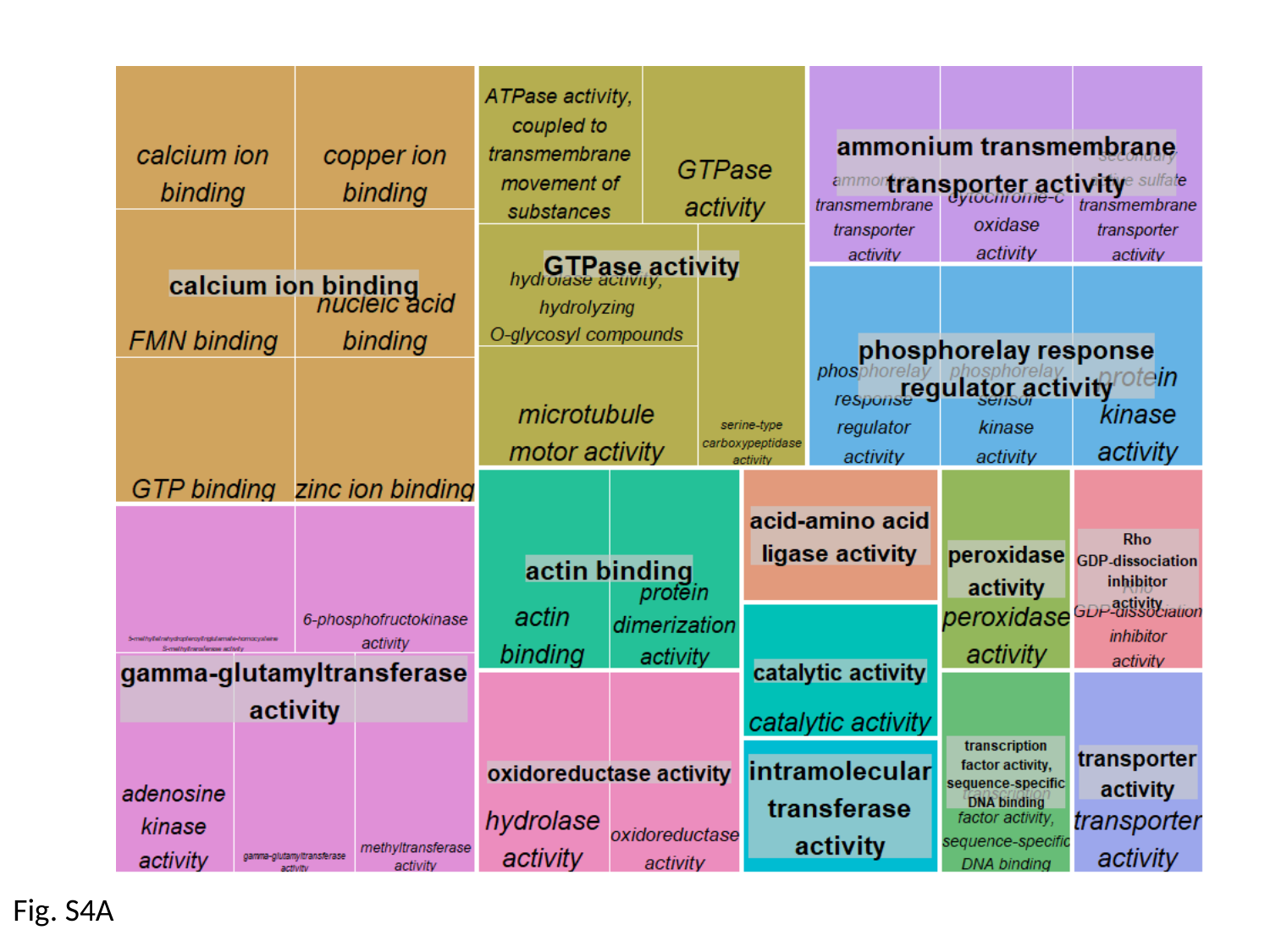

Fig. S4A

## Slide 9
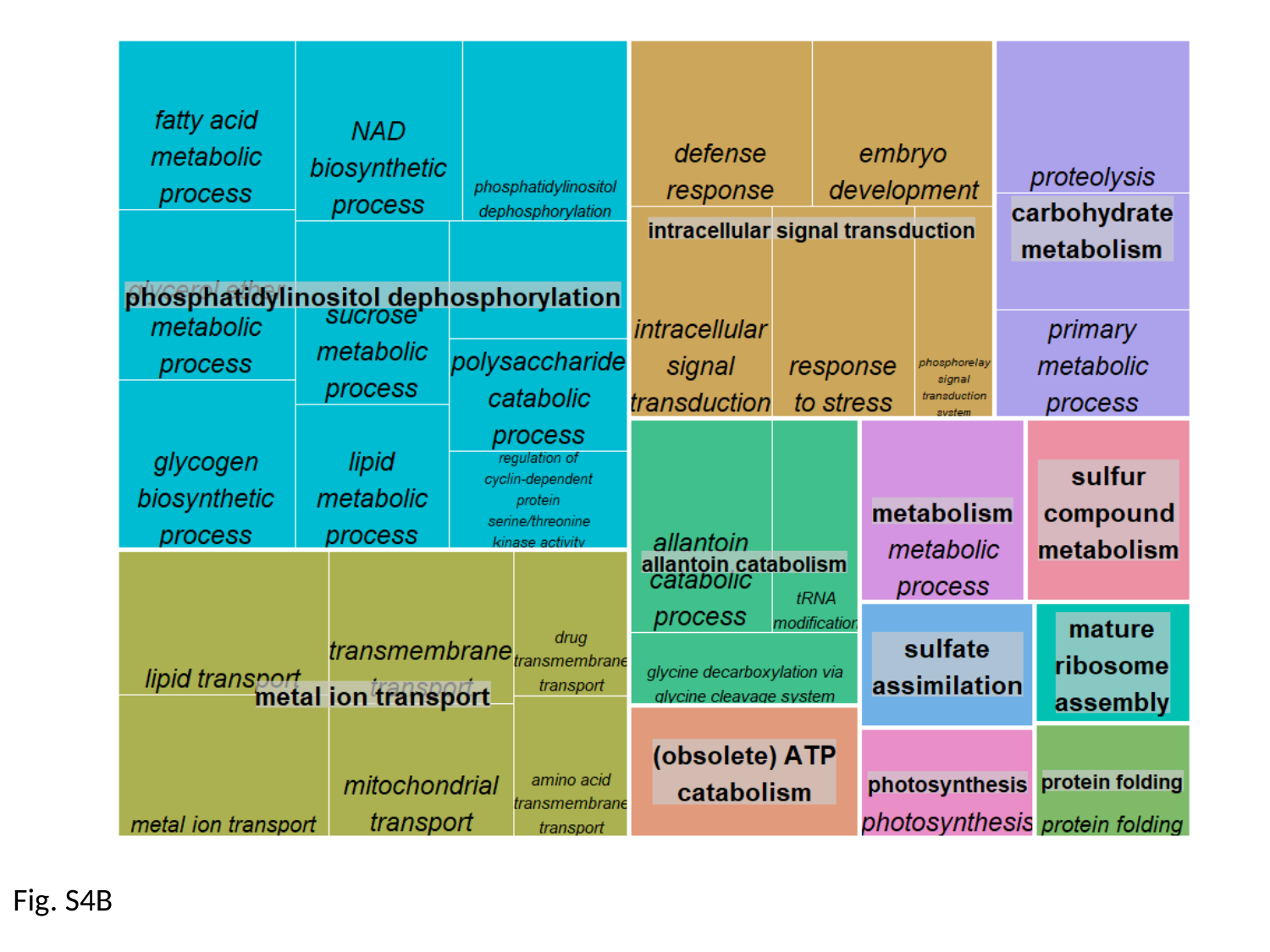

Fig. S4B

## Slide 10
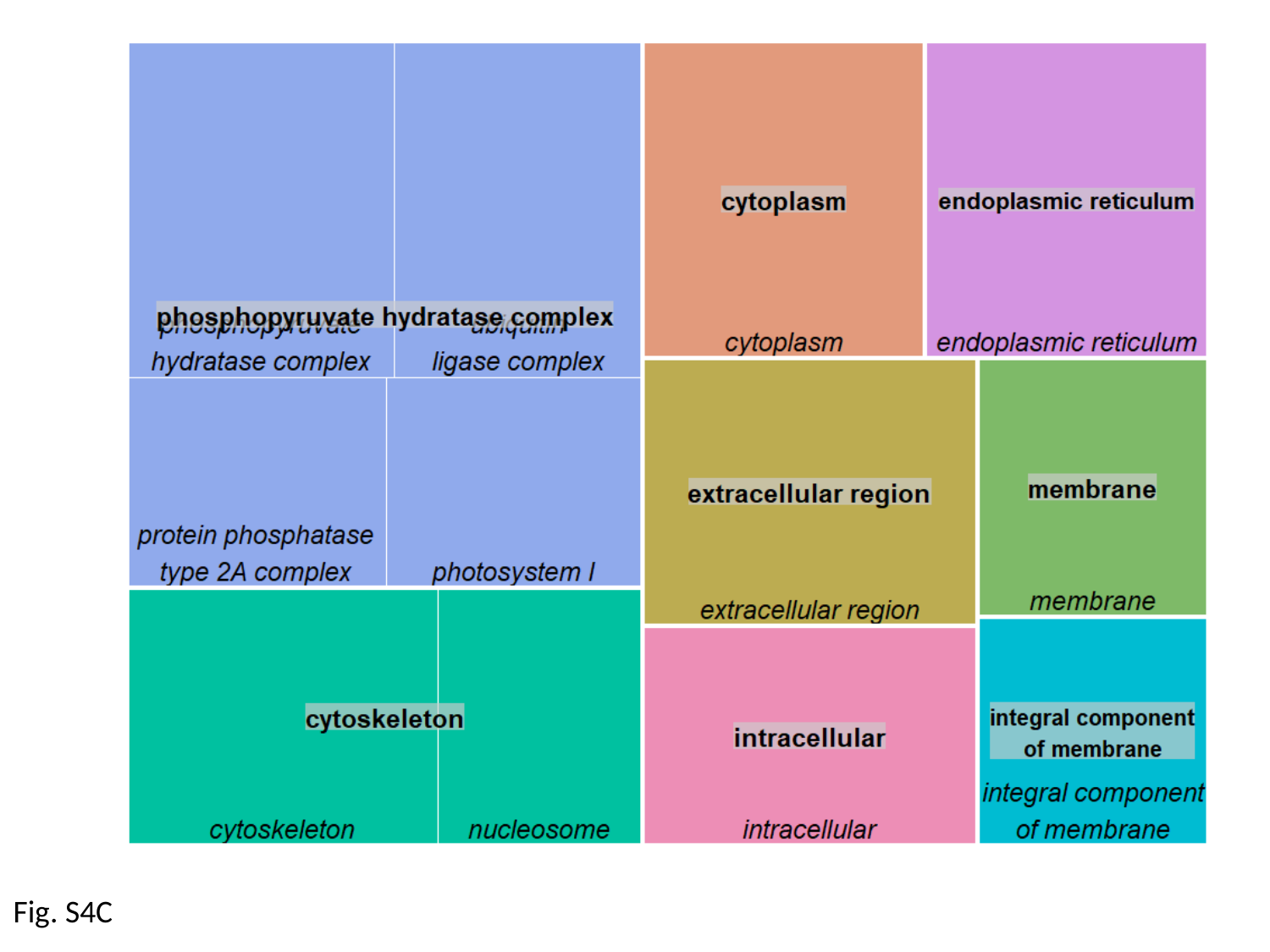

Fig. S4C

## Slide 11
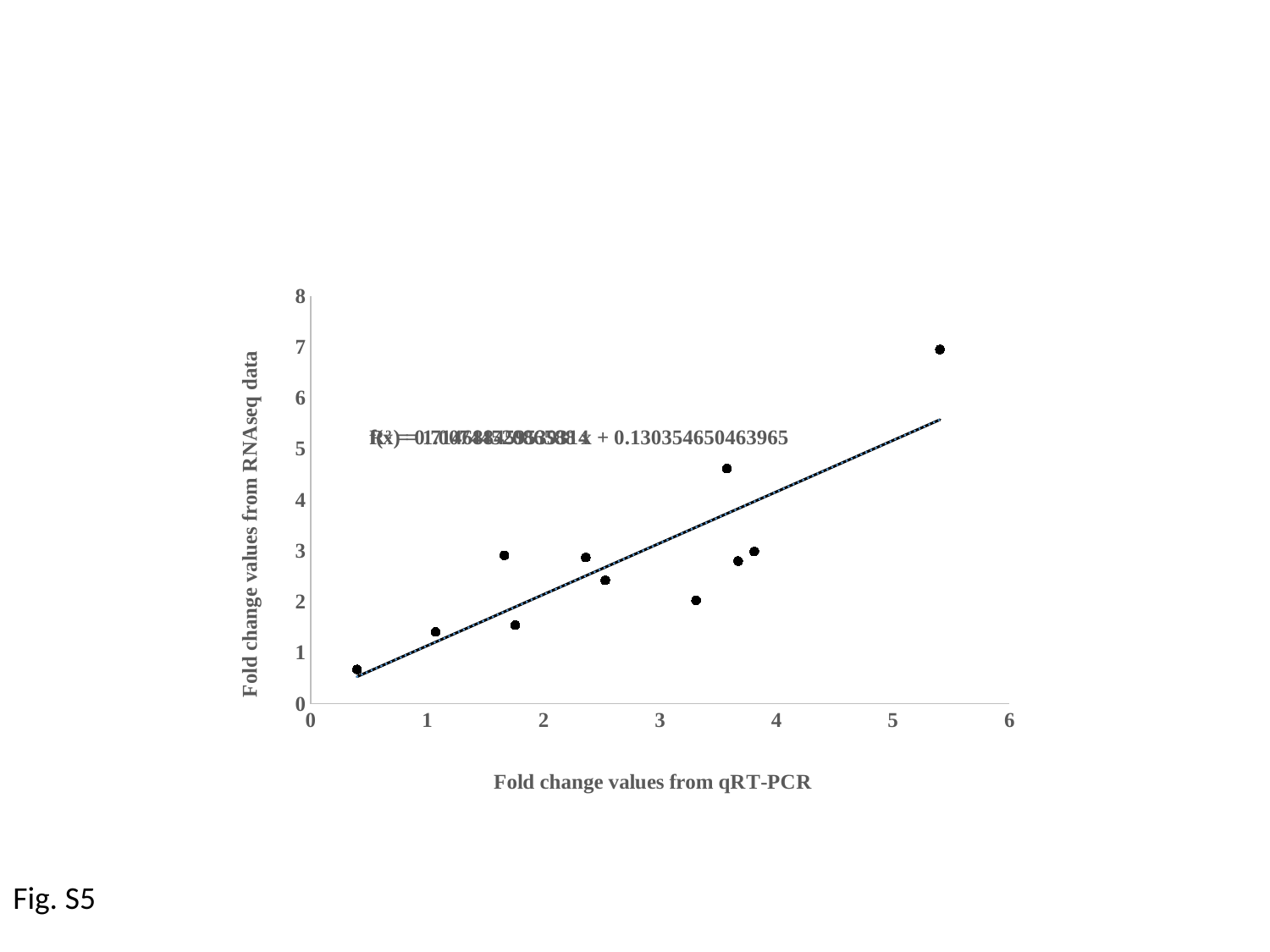

### Chart
| Category | 5.404143636 |
|---|---|Fig. S5
