# Supplementary material for: Transcriptome-IPMS analysis reveals a tissue-dependent miR156/SPL13 regulatory mechanism in alfalfa drought tolerance
Source: BMC Genomics. 2020 Oct 19;21:721. doi: 10.1186/s12864-020-07118-4 (PMC7574311; doi:10.1186/s12864-020-07118-4)
Supplement: Supplementary file 8 — Additional file 8: Table S7.. Shell scripts of Linux and R-software scripts used to analyze and visualize data. [file 12864_2020_7118_MOESM8_ESM.docx]

**Transcriptomic analysis combined with IPMS revealed tissue-dependent *miR156/SPL13* regulatory mechanism in alfalfa drought tolerance**

**Table S7. Shell scripts of Linux and R-software scripts used to analyze and visualize data**

Raw reads before trimming with sickle

**
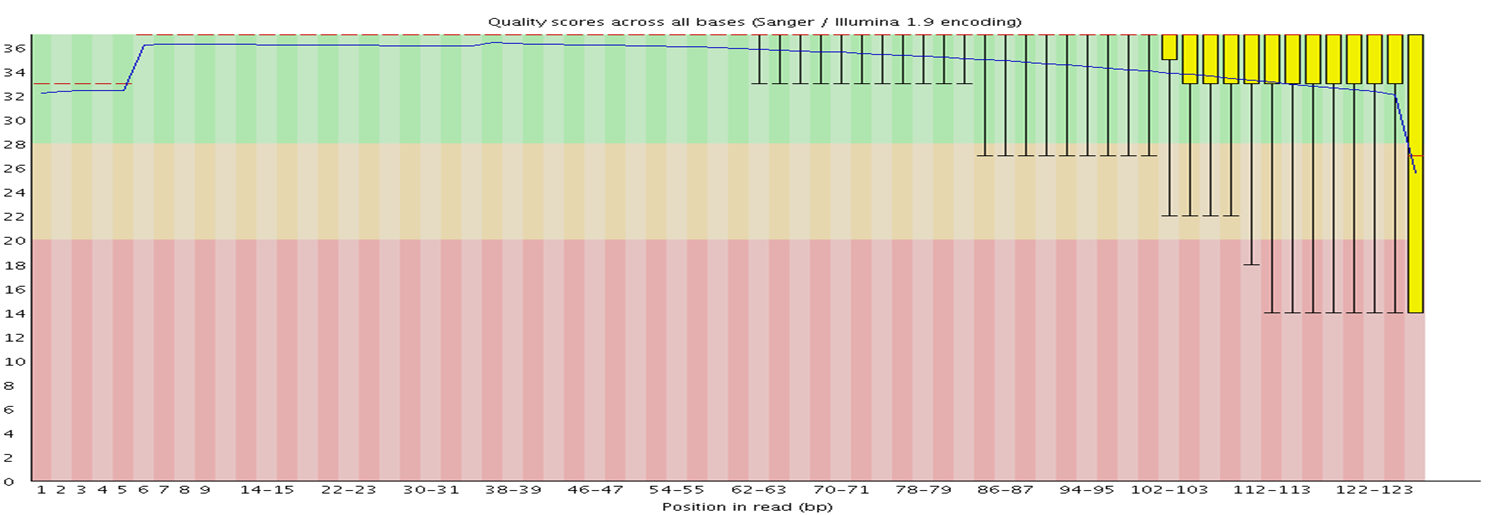
**

After trimming with sickle

**
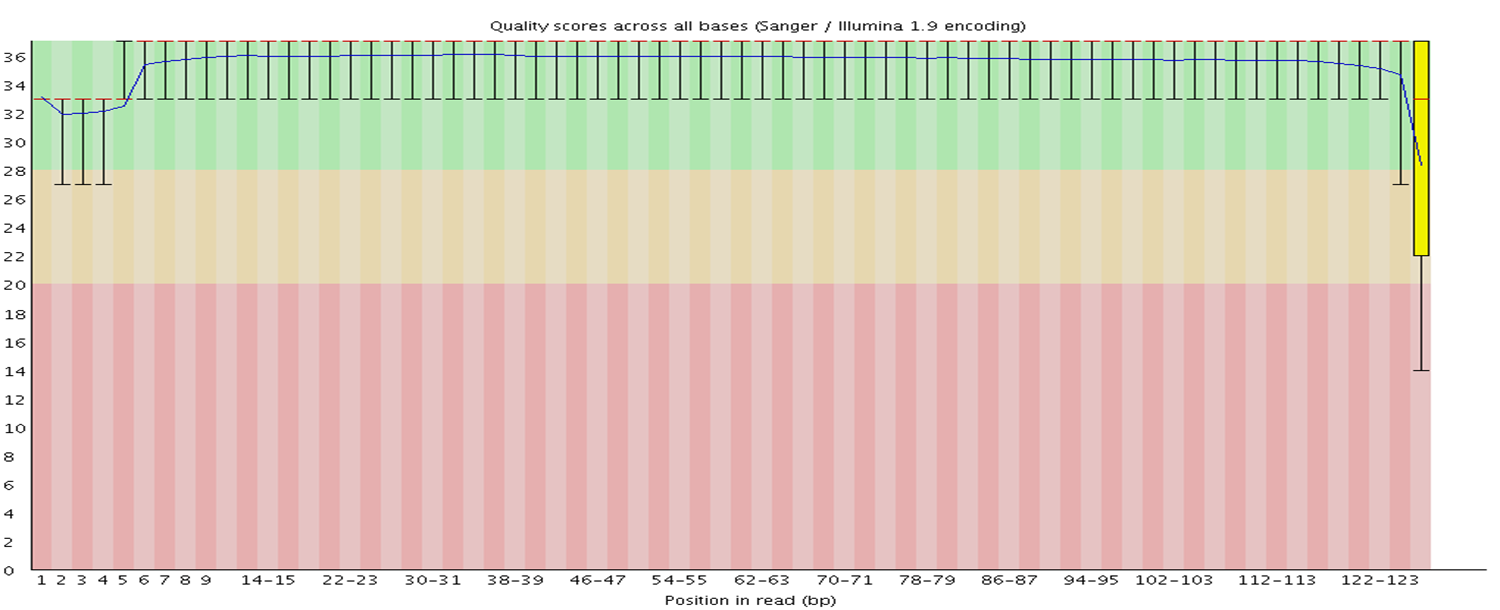
**

1 **Trimming sequence end reads with default parameters before mapping**

sickle pe -f EV-stem-trt1_R1_001.fastq -r EV-stem-trt1_R2_001.fastq -t sanger -o trimmedEV-stem-trt1_R1_001.fastq -p trimmedEV-stem-trt1_R2_001.fastq -s trimmedEV-stem-trt1.fastq

2 **Map the reads for each sample to the reference genome**

Tophat2 -p 8 -G JCVI.Medtr.v4.20130313.gtf -o EV-leaf-untrt1_thout JCVI.Medtr.v4.20130313 trimmedEV-leaf-untrt1_R1_001.fastq trimmedEV-leaf-untrt1_R2_001.fastq

3 **Assemble transcripts for each sample with reference gene structure**

cufflinks -p 8 -o ./EV-stem-trt1_clout ./EV-stem-trt1_thout/accepted_hits.bam

4 **Run Cuffmerge on all assemblies to create a single merged transcriptome annotation**

cuffmerge -g JCVI.Medtr.v4.20130313.gtf -s JCVI.Medtr.v4.20130313.fa -p 8 assemblies.txt

# assemblies.txt contains file directory paths of cufflinks generated ‘transcript.gtf’ files of all samples treated with both control and drought conditions.

5 **Run Cuffdiff by using the merged transcriptome assembly along with the BAM from Tophat for each replicate**

cuffdiff -o diff_out -b JCVI.Medtr.v4.20130313.fa -p 8 -L S13-leaf-trt,EV-leaf-trt -u merged_asm/merged.gtf S13-leaf-trt/S13-leaf-trt1_thout/accepted_hits.bam,S13-leaf-trt/S13-leaf-trt2_thout/accepted_hits.bam,S13-leaf-trt/S13-leaf-trt3_thout/accepted_hits.bam EV-leaf-trt1/EV-leaf-trt1_thout/accepted_hits.bam,EV-leaf-trt1/EV-leaf-trt2_thout/accepted_hits.bam,EV-leaf-trt1/EV-leaf-trt3_thout/accepted_hits.bam

6 **Run featureCounts to get exon read count from ‘.bam’ files generated with tophat2**

featureCounts v1.6.3; Command:featureCounts -T 8 -t exon -a Mt4.0v2_genes.gtf -o read_countnew.txt ./leaf/EV-leaf-trt1.bam ./leaf/EV-leaf-trt2.bam ./leaf/EV-leaf-trt3.bam ./leaf/EV-leaf-untrt1.bam ./leaf/EV-leaf-untrt2.bam ./leaf/EV-leaf-untrt3.bam ./leaf/S13-leaf-trt1.bam ./leaf/S13-leaf-trt2.bam ./leaf/S13-leaf-trt3.bam ./leaf/S13-leaf-untrt1.bam ./leaf/S13-leaf-untrt2.bam ./leaf/S13-leaf-untrt3.bam ./stem/EV-stem-trt1.bam ./stem/EV-stem-trt2.bam ./stem/EV-stem-trt3.bam ./stem/EV-stem-untrt1.bam ./stem/EV-stem-untrt2.bam ./stem/EV-stem-untrt3.bam ./stem/S13-stem-trt1.bam ./stem/S13-stem-trt2.bam ./stem/S13-stem-trt3.bam ./stem/S13-stem-untrt1.bam ./stem/S13-stem-untrt2.bam ./stem/S13-stem-untrt3.bam ./root/EV-root-trt1.bam ./root/EV-root-trt2.bam ./root/EV-root-trt3.bam ./root/EV-root-untrt1.bam ./root/EV-root-untrt2.bam ./root/EV-root-untrt3.bam ./root/S13-root-trt1.bam ./root/S13-root-trt2.bam ./root/S13-root-trt3.bam ./root/S13-root-untrt1.bam ./root/S13-root-untrt2.bam ./root/S13-root-untrt3.bam

**7 Differential analysis results with CummeRbund with R-software**

library(cummeRbund)

cuff_data<-readCufflinks('diff_out') #Create a CummeRbund database from the Cuffdiff output

csDensity(genes(cuff_data)) #Plot the distribution of expression levels for each sample

csScatter(genes(cuff_data), 'BvDD', 'DD') #Compare the expression of each gene in two conditions with a scatter plot

csVolcano(genes(cuff_data), 'BvDD', 'DD') #Create a volcano plot to inspect differentially expressed genes

#Exporting figures from R in Linux

png("csVolcanoBvDC_DC.png", width=1024, height=800, res=150)

csVolcano(genes(cuff_data), 'BvDC', 'DC',alpha=0.05, showSignificant=T)

graphics.off()

**8 Preparing the exon read counts for Plotting PCA with R-software**

sampleinfo <- read.delim("SampleInfo.txt", stringsAsFactors=F)

seqdata <- read.delim("read_count.txt", comment = "#", stringsAsFactors=F)

library(dplyr)

library(tibble)

newTable <- sampleinfo

basal <- which(newTable$trt=="basal")

newTable <- newTable[basal, ]

newTable <- newTable[basal, c("trt", "Sample", "tissue", "Group")]

colnames(newTable)[1] <- "trt"

countdata <- seqdata %>%

column_to_rownames("Geneid") %>% # turn the geneid column into rownames

select(sampleinfo$Sample) %>% # keep sample columns using sampleinfo$Sample

as.matrix()

keep <- rowSums(countdata) > 5

countdata <- countdata[keep,]

librarySizes <- colSums(countdata)

barplot(librarySizes,

names=names(librarySizes),

las=2,

main="Exon read count library sizes")

abline(h=7e6, lty=4)

# Get log2 counts per million

logcounts <- log2(countdata + 1)

# make a colour vector

statusCol <- as.numeric(factor(sampleinfo$Status)) + 1

# Check distributions of samples using boxplots

boxplot(logcounts,

xlab="",

ylab="Log2(exon read counts)",

las=2,

col=statusCol,

res=600)

# to alleviate the problem of rlog installation

library(ggfortify)

library(DESeq2)

rlogcounts <- rlog(countdata)

# Plotting PCA with R-software

pcDat <- prcomp(t(rlogcounts))

autoplot(pcDat)

jpeg('rplot.jpg');

p<-autoplot(pcDat,

data = sampleinfo,

fill="Group",

addEllipses = TRUE, ellipse.type = "confidence",

shape="Genotype", subtitle = 'PC1 versus PC2', vlineType = c('dotdash', 'solid', 'dashed'), hline = 0, size=5, label.size=6, width=4, height=4, units = 'in', res=480) +

scale_shape_manual(values=c(21, 24)) +

guides(fill = guide_legend(override.aes=list(shape=22)))

p + theme(panel.background = element_rect(fill = "white",

colour = "black",

size = 1, linetype = "solid"));

dev.off()

**9 Visualization of Gene Ontology-term analysis corresponding Molecular function, Biological process, and cellular components using R-software.** The script for assignment of a particular function is obtained from Revigo (http://revigo.irb.hr/) as ‘revigo.names’ followed by R-based visualization.

library(treemap)

# Run the data ‘revigo.names’ obtained from the Revigo (<http://revigo.irb.hr/>) website

stuff <- data.frame(revigo.data);

names(stuff) <- revigo.names;

stuff$abslog10pvalue <- as.numeric( as.character(stuff$abslog10pvalue) );

stuff$freqInDbPercent <- as.numeric( as.character(stuff$freqInDbPercent) );

stuff$uniqueness <- as.numeric( as.character(stuff$uniqueness) );

stuff$dispensability <- as.numeric( as.character(stuff$dispensability) );

png( file="revigo_treemap.png", width=1024, height=800, res= 150)

treemap(

stuff,

index = c("representative","description"),

vSize = "abslog10pvalue",

fontface.labels=c(2,3),

type = "categorical",

vColor = "representative",

title = "Cellular Component Gene Ontology treemap",

align.labels=list(

c("center", "center"),

c("center", "bottom")

),

inflate.labels = FALSE,

lowerbound.cex.labels = 0,

bg.labels = "#CCCCCCAA",

border.col=c("white","white"),

position.legend = "none")

dev.off()

**10. circlize, an R-software package circular data visualization**

library(circlize);

exp = read.table("leaf.txt", sep = "\t", header = T, stringsAsFactors = F);

exp_p = read.table("leaf_p.txt", sep = "\t", header = T, stringsAsFactors = F);

ss = read.table("stem.txt", sep = "\t", header = T, stringsAsFactors = F);

ss_p = read.table("stem_p.txt", sep = "\t", header = T, stringsAsFactors = F);

rr = read.table("root.txt", sep = "\t", header = T, stringsAsFactors = F);

rr_p = read.table("root_p.txt", sep = "\t", header = T, stringsAsFactors = F);

df=data.frame(exp);

dfp=data.frame(exp_p);

dfs=data.frame(ss);

dfsp=data.frame(ss_p);

dfr=data.frame(rr);

dfrp=data.frame(rr_p);

Mdtr = data.frame(

name = c("Chr1", "Chr2", "Chr3", "Chr4", "Chr5", "Chr6", "Chr7", "Chr8"),

start = c(0, 0, 0, 0, 0, 0, 0, 0),

end = c(52981375, 45728990, 55497394, 56578691, 43625231, 35241025, 49172401, 45563928))

circos.par("start.degree" = 90);

circos.genomicInitialize(Mdtr);

f=colorRamp2(breaks=c(-3, 0, 3), colors=c("blue", "white", "red"));

circos.genomicTrackPlotRegion(df, stack = TRUE,panel.fun =function(region, value, ...) {circos.genomicRect(region, value, col =f(value[[1]]),border =f(value[[1]]), ...)}, bg.border = NA, track.height = 0.075);

circos.genomicTrackPlotRegion(dfp, panel.fun =function(region, value, ...) {circos.genomicPoints(region, value, pch = 16, cex = 0.02, col="green", ...)}, track.height = 0.1);

circos.genomicTrackPlotRegion(dfs, stack = TRUE,panel.fun =function(region, value, ...) {circos.genomicRect(region, value, col =f(value[[1]]),border =f(value[[1]]), ...)}, bg.border = NA, track.height = 0.075);

circos.genomicTrackPlotRegion(dfsp, panel.fun =function(region, value, ...) {circos.genomicPoints(region, value, pch = 16, cex = 0.02, col="green", ...)}, track.height = 0.1);

circos.genomicTrackPlotRegion(dfr, stack = TRUE,panel.fun =function(region, value, ...) {circos.genomicRect(region, value, col =f(value[[1]]),border =f(value[[1]]), ...)}, bg.border = NA, track.height = 0.075);

circos.genomicTrackPlotRegion(dfrp, panel.fun =function(region, value, ...) {circos.genomicPoints(region, value, pch = 16, cex = 0.02, col="green", ...)}, track.height = 0.1);

lgd = Legend(at = c(-3, -2, 0, 2, 3), col_fun = f, title_position = "topleft", title = "Log2 FC");

draw(lgd, x = unit(3, "mm"), y = unit(3, "mm"), just = c("left", "bottom"));

circos.clear()
